# Development of DNA Vaccine Targeting E6 and E7 Proteins of Human Papillomavirus 16 (HPV16) and HPV18 for Immunotherapy in Combination with Recombinant Vaccinia Boost and PD-1 Antibody

**DOI:** 10.1128/mBio.03224-20

**Published:** 2021-01-19

**Authors:** Shiwen Peng, Louise Ferrall, Stephanie Gaillard, Chenguang Wang, Wei-Yu Chi, Chuan-Hsiang Huang, Richard B. S. Roden, T.-C. Wu, Yung-Nien Chang, Chien-Fu Hung

**Affiliations:** a Department of Pathology, The Johns Hopkins University, Baltimore, Maryland, USA; b Department of Oncology, The Johns Hopkins University, Baltimore, Maryland, USA; c Department of Obstetrics and Gynecology, The Johns Hopkins University, Baltimore, Maryland, USA; d Department of Oncology Biostatistics, The Johns Hopkins University, Baltimore, Maryland, USA; e Papivax Biotech Inc., Taipei, Taiwan, Republic of China; McMaster University

**Keywords:** human papillomavirus, HPV16, HPV18, E6, E7, DNA vaccine, HPV-associated cancer, TA-HPV, PD-1

## Abstract

Immunotherapy for cervical cancer should target high-risk human papillomavirus types 16 and 18, which cause 50% and 20% of cervical cancers, respectively. Here, we describe the construction and characterization of the pBI-11 DNA vaccine via the addition of codon-optimized human papillomavirus 18 (HPV18) E7 and HPV16 and 18 E6 genes to the HPV16 E7-targeted DNA vaccine pNGVL4a-SigE7(detox)HSP70 (DNA vaccine pBI-1). Codon optimization of the HPV16/18 E6/E7 genes in pBI-11 improved fusion protein expression compared to that in DNA vaccine pBI-10.1 that utilized the native viral sequences fused 3′ to a signal sequence and 5′ to the HSP70 gene of Mycobacterium tuberculosis. Intramuscular vaccination of mice with pBI-11 DNA better induced HPV antigen-specific CD8^+^ T cell immune responses than pBI-10.1 DNA. Furthermore, intramuscular vaccination with pBI-11 DNA generated stronger therapeutic responses for C57BL/6 mice bearing HPV16 E6/E7-expressing TC-1 tumors. The HPV16/18 antigen-specific T cell-mediated immune responses generated by pBI-11 DNA vaccination were further enhanced by boosting with tissue-antigen HPV vaccine (TA-HPV). Combination of the pBI-11 DNA and TA-HPV boost vaccination with PD-1 antibody blockade significantly improved the control of TC-1 tumors and extended the survival of the mice. Finally, repeat vaccination with clinical-grade pBI-11 with or without clinical-grade TA-HPV was well tolerated in vaccinated mice. These preclinical studies suggest that the pBI-11 DNA vaccine may be used with TA-HPV in a heterologous prime-boost strategy to enhance HPV 16/18 E6/E7-specific CD8^+^ T cell responses, either alone or in combination with immune checkpoint blockade, to control HPV16/18-associated tumors. Our data serve as an important foundation for future clinical translation.

## Abstract (precis)

Persistent expression of high-risk human papillomavirus (HPV) E6 and E7 is an obligate driver for several human malignancies, including cervical cancer, wherein HPV16 and HPV18 are the most common types. PD-1 antibody immunotherapy helps a subset of cervical cancer patients, and its efficacy might be improved by combination with active vaccination against E6 and/or E7.

## INTRODUCTION

Human papillomavirus (HPV) is a common etiological factor in several human cancers, including cervical, anal, penile, vulvar, vaginal, and head and neck cancers (1). High-risk human papillomavirus (hrHPV) infections, such as HPV16 and HPV18, cause approximately 5% of all cancer cases globally (2), including >95% of all cervical cancers (3–5). Approximately 50% of cervical cancer is related to HPV16, and ∼20% is related to HPV18 (5). Approximately 65% of all head and neck squamous cell carcinomas are HPV positive, with HPV16 in >90% of HPV-associated oropharyngeal squamous cell carcinoma cases (6–10). Due to the high burden of HPV-associated cancers, there is significant interest in developing treatment options. While the licensed HPV vaccines, e.g., Gardasil and Cervarix, prevent HPV infection, they do not clear existing infections. There are no therapeutic options for individuals with persistent hrHPV infection beyond continued screening. High-grade squamous intraepithelial lesions, the precursors of cervical cancer, require surgical intervention to remove or other interventions to ablate the lesion (11). Although some HPV-associated cancers can be treated in their early stages, for patients with metastatic or advanced HPV-associated cancers, therapeutic options have limited success and can come with significant morbidity. Therapeutic HPV vaccines represent a promising alternative treatment strategy to clear high-risk HPV infections and associated malignancies.

E6 and E7 are common immunotherapeutic targets for the development of vaccines against HPV-associated cancers (12) for several key reasons. First, E6 and E7 are consistently expressed in HPV-associated malignancies and HPV-infected cells, but not in healthy cells, providing specificity (13, 14). Second, E6/E7 oncogenic proteins are typically required for the initiation and maintenance of HPV-associated malignancies, rendering them essential for cancer growth and preventing immune escape (15). Third, E6/E7 oncogenic proteins are foreign viral antigens and are not subject to central tolerance by human immune systems. Several forms of therapeutic HPV vaccines have been developed to target HPV E6 and/or E7. These therapeutic HPV vaccines include viral vector or bacterial vector-based, nucleic acid-based, peptide/protein-based, or cell-based vaccines (for review, see reference 12). One such vaccine, tissue-antigen human papillomavirus vaccine (TA-HPV), is a live recombinant vaccinia virus vector-based vaccine that expresses HPV16 and HPV18 E6-E7 fusion proteins (16). TA-HPV has been tested in several early-phase clinical trials in patients with cervical cancer (17, 18) and high-grade cervical (19) and anogenital intraepithelial neoplasia (20, 21); although well tolerated, it showed little evidence of clinical benefit. Preclinical studies showed the HPV antigens are subdominant in the context of TA-HPV, demonstrating the importance of heterologous prime-boost vaccination for optimal immunogenicity (22) and the potential for use in conjunction with immunomodulating approaches to address immune suppression mechanisms within the tumor (for review, see reference 23).

DNA vaccines offer stability, safety, and simplicity of manufacture, and they can be administered multiple times. However, intramuscular DNA vaccination alone has limited potency, but it has been used to prime the immune response prior to administration with a replication-competent viral vector-based vaccine such as TA-HPV (22). In particular, DNA vaccination with HPV E6 and E7 is poorly immunogenic, likely because the virus has evolved mechanisms to evade host recognition, including low levels of E6/E7 expression. As a result, several strategies to circumvent this limited expression and enhance potency have been developed. For example, the Mycobacterium tuberculosis heat shock protein 70 (HSP70) has been linked to HPV E7 antigen in a DNA vaccine plasmid to enhance immunogenicity (24). Mycobacterium tuberculosis HSP70 provides an alarmin-like function that delivers linked proteins to the dendritic cell, thereby facilitating the presentation of the antigen by major histocompatibility complex (MHC) class I via cross-presentation (25). Vaccination with DNA expressing E7 fused to M. tuberculosis HSP70 robustly increases E7-specific CD8^+^ T cell responses and therapeutic antitumor effects against the E7-expressing TC-1 tumor compared to responses from vaccination with E7 DNA, HSP70 DNA, or the combination (24, 26–29). Addition of a signal peptide (Sig) for secretion of the linked E7-HSP70 fusion protein and enhanced cross-presentation of E7 by antigen-presenting cells further increases the CD8^+^ T cell responses and therapeutic antitumor effects (22, 28, 30–32). The pNGVL4a-Sig/E7(detox)/HSP70 DNA vaccine (here termed pBI-1) encodes a fusion protein consisting of the Mus musculus LAMP-1 signal peptide fused in frame to HPV16 E7(detox), which has a point mutation that eliminate its oncogenic potential, and Mycobacterium tuberculosis HSP70 (28).

**FIG 1:**
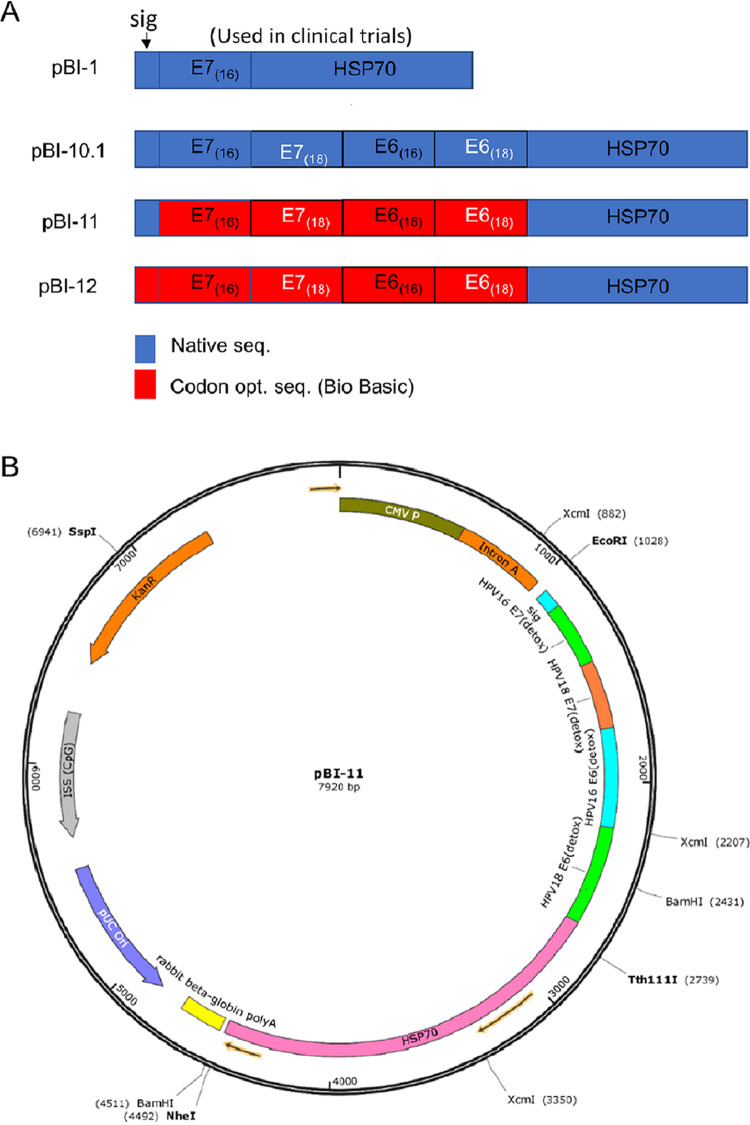
Schematic of the DNA constructs and map of pBI-11. (A) Schematic of the DNA constructs that are cloned into the pNGVL4a plasmid. The genes in the plasmids pBI-1, pBI-10.1, pBI-11, and pBI-12 are depicted. pBI-1 is in use for clinical trials under the name pNGVL4a-Sig/E7(detox)/HSP70. Blue indicates the native sequence of the target antigen. HSP70 is derived from Mycobacterium tuberculosis. Red indicates the gene was codon optimized prior to cloning it into the vector. (B) DNA construct map of pBI-11.

In several clinical trials, vaccination with pBI-1 was well tolerated (19, 30), including as a priming immunization prior to boosting with TA-HPV (19). In a small study, half of the women with HPV16^+^ cervical intraepithelial neoplasia grade 2/3 (CIN2/3) demonstrated complete histologic clearance after intramuscular vaccination twice with 3 mg of pBI-1 DNA followed by 10^7^ PFU of TA-HPV administered at monthly intervals (19). As a priming vaccination for TA-HPV, pBI-1 is suboptimal because it only targets HPV16 E7, whereas TA-HPV expresses E6 and E7 of both HPV16 and HPV18. This suggests that using a DNA vaccine that is based on pBI-1 but targets all four HPV oncoproteins could improve the breadth and clinical efficacy of this immunotherapy approach. We therefore constructed three vaccines using the pBI-1 backbone that included all four antigens with different codon optimization schemes (Fig. 1). Codon optimization has the potential to enhance immunogenicity of DNA vaccines by increasing the expression of the encoded antigen (for review, see reference 33).

Tumor cells often develop mechanisms to subvert or overcome spontaneous or induced antitumor immunity. For example, upregulation of PD-L1 on the surface of tumor cells engages the PD-1 receptor on immune cells, triggering their apoptosis and thus blunting the preexisting immune response. Blockade of this immune checkpoint signaling can help overcome immunosuppression. PD-1 antibody blockade improves outcomes for a fraction of cervical cancer patients (for review, see reference 34). Failure of this treatment may be due to several factors, such as absent expression of PD-L1/PD-1 or a poor immune response (35). However, the best response to this immunotherapy is associated with a robust prior CD8^+^ T cell infiltrate (36, 37). Combining PD-1 antibody treatment with prior or concomitant therapeutic vaccination may therefore improve the overall antitumor response. Here, we examine the potential for application of PD-1 blockade in conjunction with a DNA prime and TA-HPV vaccinia boost vaccination regimen for the control of advanced HPV-associated cancers.

## RESULTS

### Codon optimization leads to increased expression of the encoded HPV antigen.

**FIG 2:**
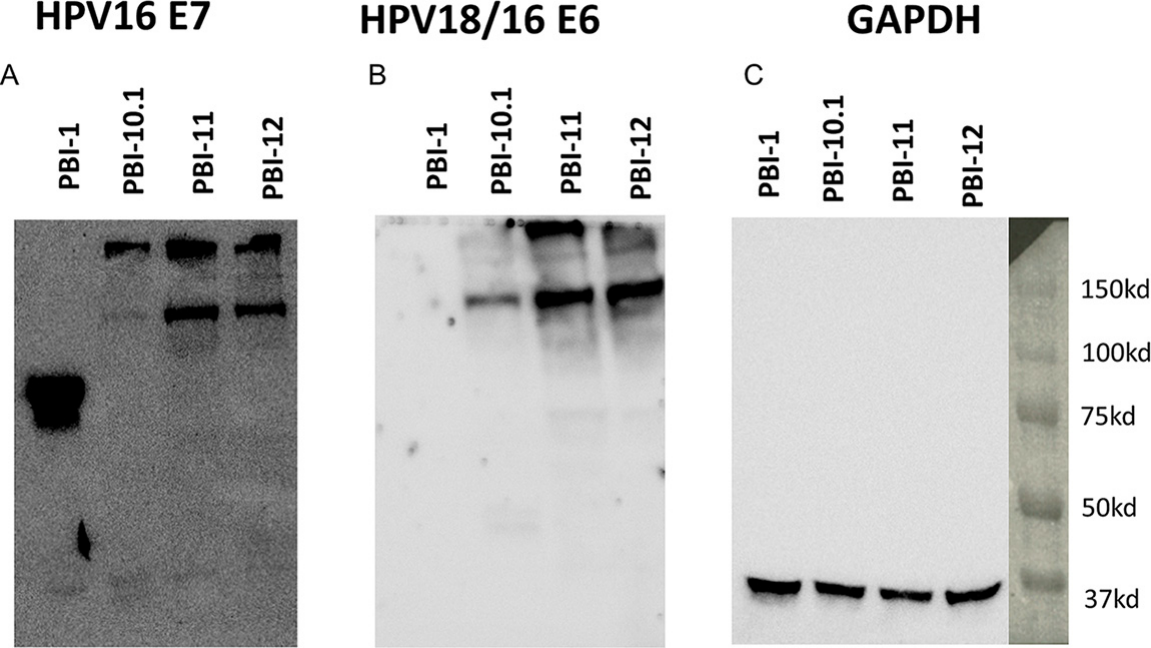
Western blot analysis to characterize the expression of the encoded HPV16/18 E6/E7 fusion protein in transfected cells. 293 expi cells were transfected with the designated plasmids and analyzed by Western blotting to assess the levels of target antigens. HPV16 E7-specific (A) and HPV18 and HPV16 E6-specific (B) MAbs were used as probes for the Western blot analysis of the antigen expression of the DNA vaccine constructs. 293 cells were transfected with the vaccines. (C) GAPDH was used as the loading control.

To modify the pBI-1 DNA vaccine to also target HPV18 E7 and both HPV16 and HPV18 E6, we generated four different DNA constructs (Fig. 1). While pBI-1 included only a non-codon-optimized (native) sequence for HPV16 E7 antigen, pBI-10.1 comprised native sequences encoding HPV16 E6/E7 and HPV18 E6/E7. pBI-11 used codon-optimized sequences for HPV16 E6/E7 and HPV18 E6/E7 antigens, and in pBI-12, the signal peptide was also codon optimized (Fig. 1A). Figure 1B shows the plasmid map of codon-optimized pBI-11. To determine whether cells transfected with the various DNA constructs appropriately produce the encoded fusion protein, 293 expi cells were transfected, and after 1 day, total cell lysates were harvested. An HPV16 E7-specific monoclonal antibody was used to characterize the expression of the fusion protein by Western blotting. The blots were stripped and reprobed with a monoclonal antibody targeting both HPV16 E6 and HPV18 E6. Finally, the blot was stripped and reprobed with an antibody to glyceraldehyde-3-phosphate dehydrogenase (GAPDH) as a loading control (Fig. 2).

All four vaccines resulted in the expression of HPV16 E7 antibody-reactive fusion protein (Fig. 2A). The similar band sizes of antigens produced by the pBI-10.1, pBI-11, and pBI-12 DNA vaccines show that they made identical antigen, whereas the differing intensities of the bands suggested different antigen expression levels. Addition of the native sequences for HPV18 E7 and HPV16/18 E6 in pBI-10.1 was associated with suppressed expression of the fusion protein. However, codon optimization of HPV18 E7 and HPV16/18 E6 in pBI-11 and pBI-12 resulted in robust expression of the fusion protein, although less than that for pBI-1 (Fig. 2B). Densitometry analysis of the blots further confirmed that HPV16/18 E6 antigen was expressed in larger amounts following vaccination by pBI-11 (3.7×) and pBI-12 (2.8×) than by pBI-10.1 (1×). Further codon optimization of the signal peptide in pBI-12 failed to further enhance expression over that from pBI-11 (Fig. 2B). This was confirmed by probing the Western blot using an HPV16/18 E6-specific antibody; however, as pBI-1 does not encode E6 antigen, no reactivity was detected in cells transfected with pBI-1. In comparison, HPV16/18 E6 was detected in all of the cells transfected with pBI-10.1, pBI-11, or pBI-12. Similar to that found for the E7-specific Western blot, pBI-11 and pBI-12 had enhanced expression levels of E6 compared to that for pBI-10.1 (Fig. 2B). Using GAPDH as the loading control (Fig. 2C), the blots suggest that codon optimization of all four HPV genes in pBI-11 and pBI-12 leads to improved expression of the encoded protein but at a level lower than that seen for pBI-1.

### Cells transfected with DNA constructs with codon-optimized HPV genes have enhanced presentation of HPV antigenic peptide by MHC class I molecules.

**FIG 3:**
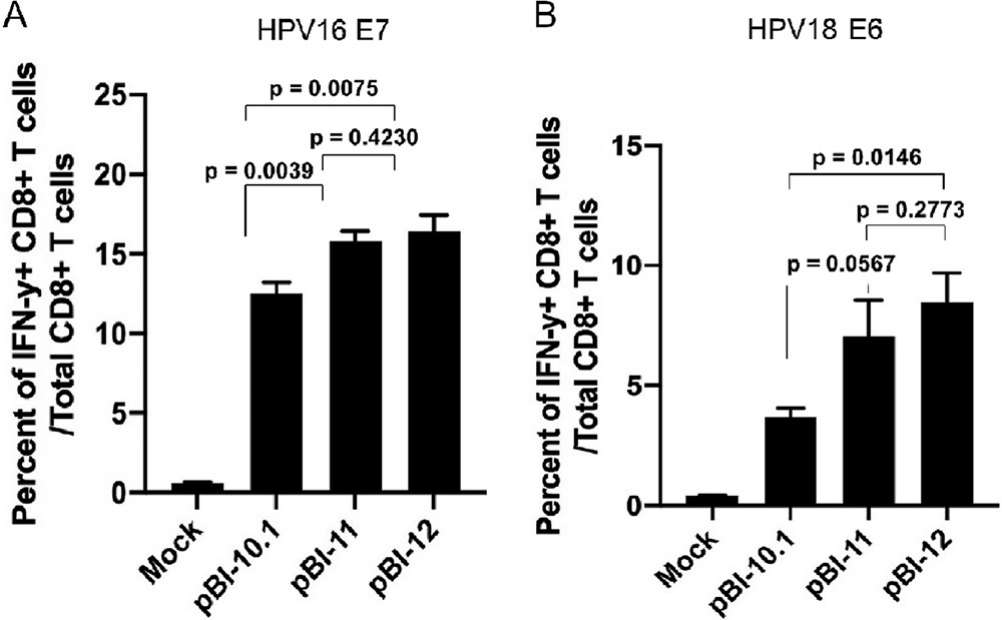
Characterization of HPV16 E7 and HPV18 E6 antigen presentation by cells transfected with the various DNA vaccines. 293-D^b^ or 293 K^b^ cells were transfected with either pBI-10.1, pBI-11, or pBI-12 DNA vaccine or mock transfected using Lipofectamine 2000. Twenty-four hours later, these cells were harvested and cocultured with either murine H-2D^b^-restricted HPV16 E7 (aa 49 to 57) peptide-specific CD8^+^ T cells or murine H-2K^b^-restricted HPV 18 E6 (aa 67 to 75) peptide-specific CD8^+^ T cells in the presence of GolgiPlug. The cells were then harvested, and IFN-γ intracellular staining was performed to determine the activation of HPV16 E7 or HPV18 E6 antigen-specific CD8^+^ T cells. (A) Bar graph to summarize flow cytometry data for activation of HPV16 E7-specific CD8^+^ T cells. (B) Bar graph to summarize the flow cytometry data for the activation of HPV18 E6-specific CD8^+^ T cells.

To compare the capacity of HPV antigen presentation by cells transfected with either pBI-10.1, pBI-11, or pBI-12 DNA vaccine, we performed *in vitro* T cell activation assays using murine H-2D^b^-restricted HPV16 E7 peptide (amino acids [aa] 49 to 57)-specific CD8^+^ T cells (Fig. 3A) or H-2K^b^-restricted HPV18 E6 peptide (aa 67 to 75)-specific CD8^+^ T cells (Fig. 3B). Mock-transfected cells were used as the control. As shown in Fig. 3, cells transfected pBI-11 or pBI-12 were able to activate more HPV 16 E7 peptide (aa 49 to 57)-specific CD8^+^ T cells and HPV 18 E6 peptide (aa 67 to 75)-specific CD8^+^ T cells than cells transfected with pBI-10.1. Mock-transfected cells did not have any appreciable activation of E7 peptide (aa 49 to 57)-specific CD8^+^ T cells. These data indicate the enhanced expression of HPV 16/18 E6/E7 fusion proteins by codon optimization in the DNA vaccine facilitate the presentation of HPV antigens by MHC class I molecules to activate HPV antigen-specific CD8^+^ T cells.

### Codon optimization of the DNA construct leads to enhanced HPV16 E7-specific CD8^+^ T cell-mediated immune responses.

**FIG 4:**
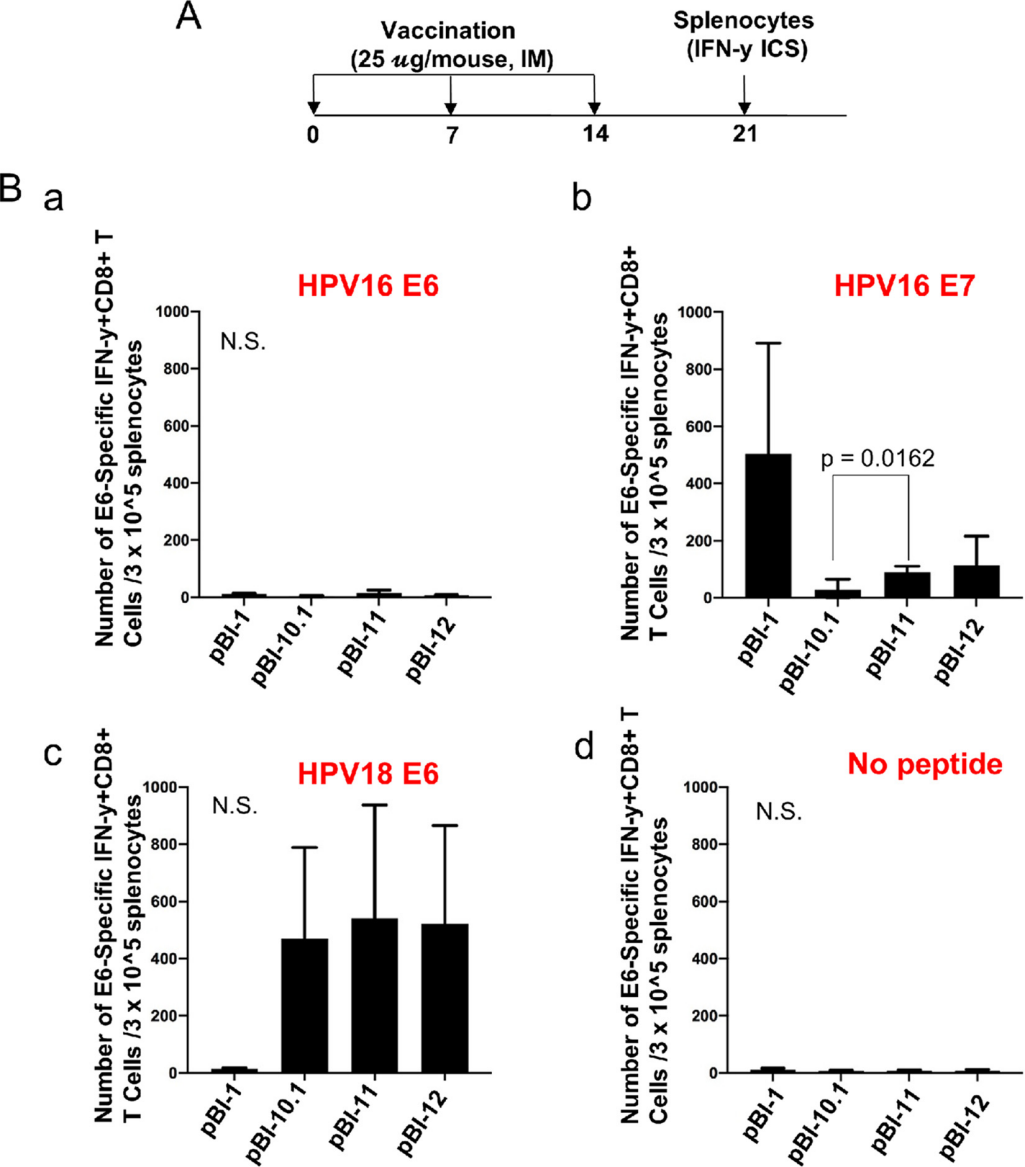
Comparison of HPV16/18 E6/E7-specific CD8^+^ T cell responses generated by the various HPV DNA vaccines. (A) Schematic illustration of the experimental design. Briefly, 5- to 8-week-old female C57BL/6 mice (5 mice/group) were vaccinated with either 25 μg/mouse of pBI-10.1, pBI-11, or pBI-12 DNA vaccine through intramuscular injection. The mice were boosted twice with the same dose and regimen with 1-week intervals. Seven days after the last vaccination, splenocytes were prepared from the vaccinated mice and stimulated with HPV16 E7 (aa 49 to 57) peptide, HPV16 E6 (aa 50 to 57) peptide, or HPV18 E6 (aa 67 to 75) peptide in the presence of GolgiPlug overnight. The splenocytes were stained with PE-conjugated anti-mouse CD8a. After permeabilization and fixation, the cells were further stained with FITC-conjugated anti-mouse IFN-γ. The cells were acquired with a FACSCalibur flow cytometer, and data were analyzed with CellQuest Pro software. (B) Bar graphs summarizing the data from flow cytometry analysis of HPV16 E6 (a), HPV16 E7 (b), and. HPV18 E6 (c) peptide-specific CD8^+^ T cell responses analyzed by IFN-γ intracellular staining. (d) Splenocytes pulsed without peptide as negative control. N.S., not significant.

To assess the impact of targeting four HPV genes and use of codon optimization, an immunogenicity study was performed in naive mice. C57BL/6 mice were utilized because extensive CD8 T cell epitope mapping within the four oncoproteins has been performed and three principle epitopes defined, namely, HPV18 E6 aa 67 to 75 (38), HPV16 E7 aa 49 to 57, and HPV16 E6 aa 50 to 57 (39), in descending order of strength. Therefore, 6- to 8-week-old female C57BL/6 mice were vaccinated with 25 μg/mouse of either pBI-1, pBI-10.1, pBI-11, or pBI-12 plasmid intramuscularly 3 times at 7-day intervals. One week after the last vaccination, splenocytes were collected (Fig. 4A) for analysis of CD8^+^ T cell response as determined by intracellular cytokine staining for interferon γ followed by flow cytometry after stimulation with the known MHC-I peptides in HPV16 E6 and E7 and HPV18 E6. The pBI-10.1-vaccinated mice had a significantly weaker E7-specific CD8^+^ T cell-mediated immune response than codon-optimized pBI-11-vaccinated mice (*P* = 0.0162), and pBI-12 showed no significant improvement compared to that with pBI-11 (Fig. 4Bb). pBI-1 elicited the highest HPV16 E7-specific response (Fig. 4Bb). Based on the fusion protein expression levels (Fig. 2), this suggests that the enhanced expression of antigen in pBI-11 compared to that in pBI-10.1 due to codon optimization translates to more potent T cell-mediated immune responses to E7 in vaccinated mice. We also characterized the HPV18 E6-specific CD8^+^ T cell-mediated immune responses. Mice vaccinated with pBI-1 DNA vaccine were used as negative controls, as the pBI-1 plasmid does not include a gene encoding an E6 antigenic protein. As shown in Fig. 4Bc, all of the DNA constructs, including pBI-10.1, pBI-11, and pBI-12, generated potent HPV 18 E6-specific CD8^+^ T cell-mediated immune responses. In contrast, vaccination with pBI-1 failed to generate detectable HPV18 E6-specific CD8^+^ T cell-mediated immune responses. The pBI-11 and pBI-12 DNA-vaccinated mice displayed slightly higher numbers of HPV18 E6-specific CD8^+^ T cells, although it was not statistically significant. Our data suggest that the HPV18 E6 peptide (aa 67 to 75)-specific CD8^+^ T cell-mediated immune response is dominant compared to the HPV16 E7 peptide (aa 49 to 57)-specific CD8^+^ T cell-mediated immune responses, and immune competition may also contribute to the lower HPV16 E7-specific response in the pBI-10.1-, pBI-11-, and pBI-12 DNA-vaccinated mice than in mice vaccinated with pBI-1. The HPV16 E6 epitope is known to be weak, and no significant response was detected (Fig. 4Ba). Our data indicated that while the codon-optimized DNA vaccine is able to lead to significantly enhanced HPV16 E7-specific CD8^+^ T cell-mediated immune responses, this difference was not evident for HPV18 E6-specific CD8^+^ T cell-mediated immune responses in naive C57BL/6 mice. Additionally, the benefit of codon optimization of Sig in pBI-12 is negligible.

### Codon-optimized pBI-11/pBI-12 DNA vaccination elicits a therapeutic antitumor response in an HPV16 E6/E7 expression tumor model, TC-1.

**FIG 5:**
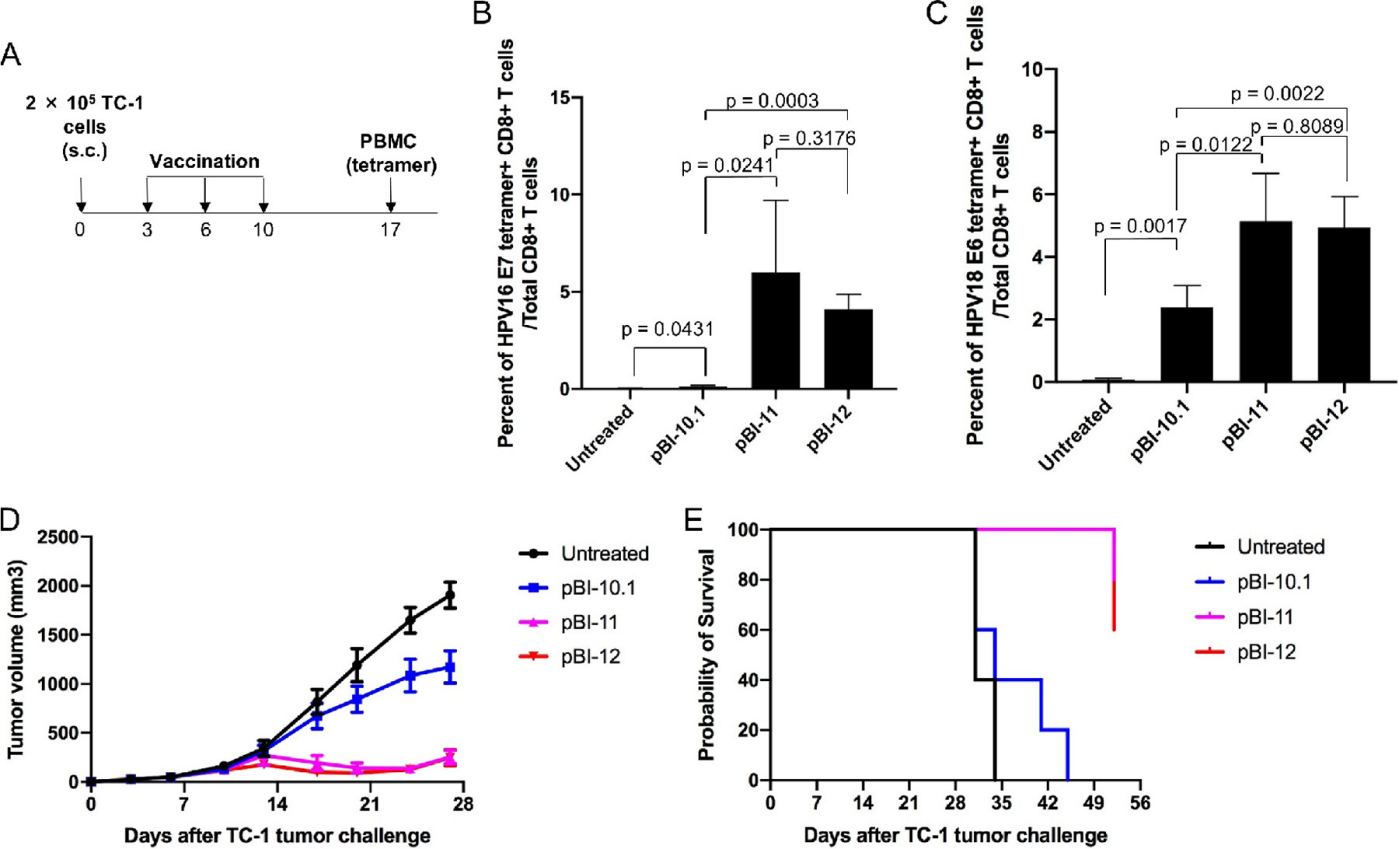
Characterization of HPV antigen-specific CD8^+^ T cell-mediated immune responses and therapeutic antitumor effects of the various DNA vaccines in mice injected with HPV16 E6/E7-expressing tumors, model TC-1. Six- to eight-week-old female C57BL/6 mice (5 mice/group) were injected with 2 × 10^5^ of TC-1 cells subcutaneously on day 0. On day 3, the mice were vaccinated with either pBI-10.1, pBI-11, or pBI-12 DNA (25 μg/50 μl/mouse) through i.m. injection and boosted twice as indicated or left untreated as control. (A) Schema of the experiment. (B) Detection of HPV16 E7-specific CD8^+^ T cells in peripheral blood using HPV16 E7 (aa 49 to 57) peptide-loaded tetramer staining. (C) Detection of HPV18 E6-specific CD8^+^ T cells in peripheral blood using HPV18 E6 (aa 67 to 75) peptide-loaded tetramer staining. (D) Summary of the TC-1 tumor volumes of the mice. (E) Kaplan-Meier analysis of the survival of TC-1 tumor-bearing mice.

We further examined the ability of the various DNA vaccines to generate therapeutic antitumor effects against the HPV16 E6/E7^+^ TC-1 tumor model. Thus, 6- to 8-week-old female C57BL/6 mice (5 mice/group) were injected with 2 × 10^5^ TC-1 cells subcutaneously on day 0. On day 3, the mice were either treated with each of the DNA vaccines (25 μg in 50 μl/mouse) through intramuscular (i.m.) injection at the hind legs and boosted at the same dose and regimen twice at a 1-week interval. Mice left untreated were used as a control. One week after the final injection, peripheral blood mononuclear cells (PBMCs) were collected (Fig. 5A). Tumor-bearing mice treated with pBI-11 or pBI-12 had significantly higher percentages of HPV16 E7-specific CD8^+^ T cell-mediated immune responses than mice vaccinated with pBI-10.1 (Fig. 5B). Likewise, tumor-bearing mice treated with pBI-11 or pBI-12 generated significantly higher percentages of HPV18 E6-specific CD8^+^ T cell-mediated immune responses than mice treated with pBI-10.1 (Fig. 5C). The stronger immune responses generated by codon optimization in the DNA vaccines translates into more potent antitumor efficacy. As shown in Fig. 5D, tumors in mice vaccinated with pBI-11 or pBI-12 grew significantly slower than in mice treated with pBI-10.1 (Fig. 5D). Likewise, vaccination with pBI-11 and pBI-12 resulted in better survival that that for mice treated with pBI-10.1 or untreated mice (Fig. 5E), although all eventually succumbed to the tumor by day 52. This suggests that antigen expression is limiting such that improved fusion protein expression through codon optimization enhances the antitumor immune response against established TC-1 tumors to prolong survival, but it was still not sufficient to cure the mice in this context. There was no significant difference between pBI-11 and pBI-12, further confirming previously discussed results that optimization of Sig in pBI-12 does not significantly enhance either expression or immunogenicity. Notably, despite not being codon optimized and producing low levels of HPV antigen, pBI-10.1 did trigger a significant immune response.

### HPV antigen-specific CD8^+^ T cell-mediated immune responses generated by the pBI-11 DNA vaccine can be further enhanced by boost with TA-HPV vaccinia virus vaccine.

**FIG 6:**
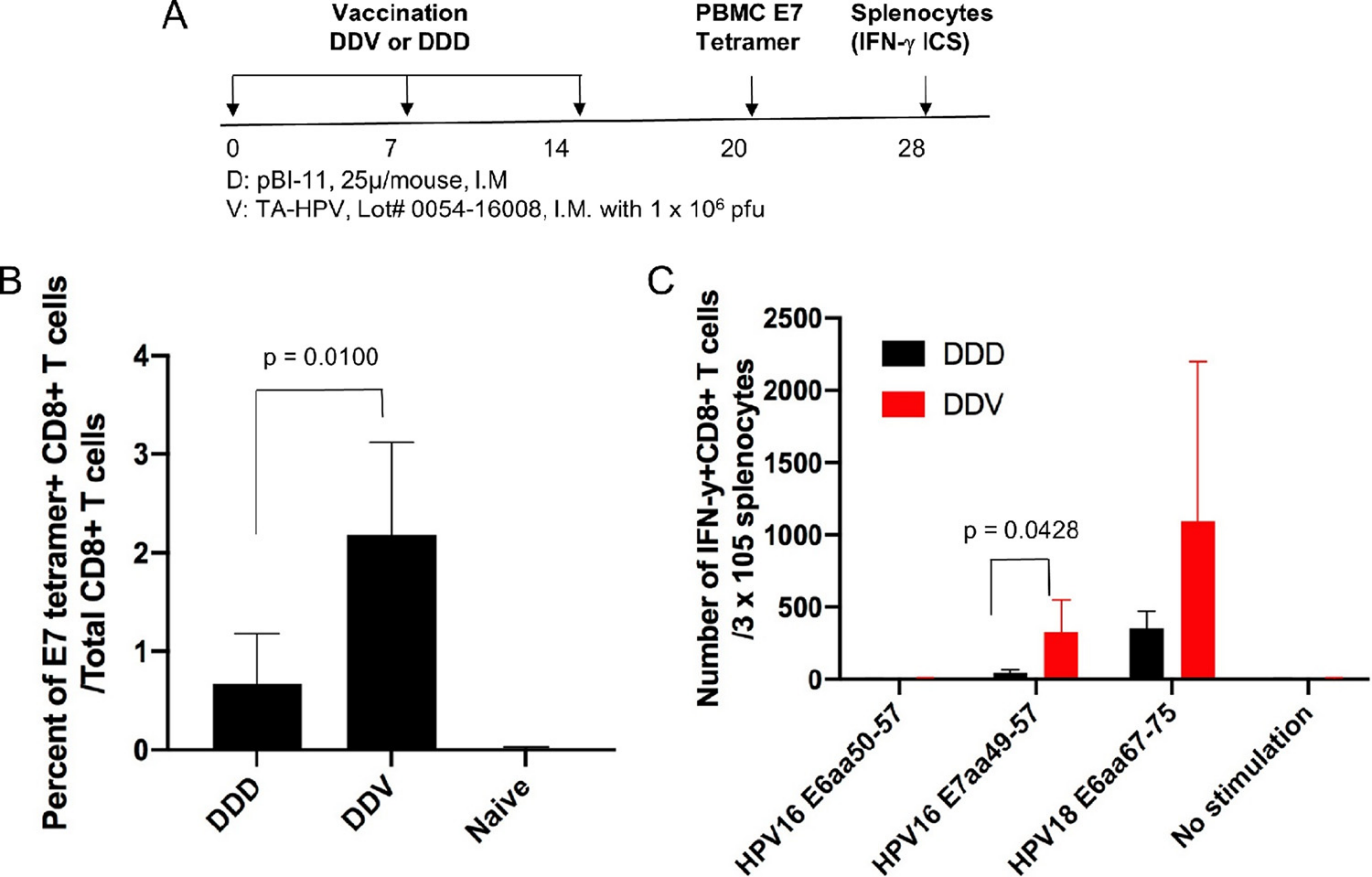
Characterization of HPV antigen-specific CD8^+^ T cell-mediated immune responses in mice vaccinated with pBI-11 DNA alone or followed by TA-HPV. Six- to eight-week-old female C57BL/6 mice (5 mice/group) were vaccinated with pBI-11 DNA (25 μg/50 μl/mouse) through i.m. injection. The mice were boosted with the same regimen 7 days later. One week after the second vaccination, one group of the mice was vaccinated with pBI-11 DNA (25 μg/50 μl/mouse) through i.m. injection (DDD). Another group of mice was vaccinated with TA-HPV (1 × 10^6^ PFU/50 μl/mouse) through i.m. injection (DDV). (A) Schema of the experiment. Six days after the last vaccination, peripheral blood was collected from the vaccinated or naive mice for HPV16 E7 tetramer staining. Fourteen days after the last vaccination, splenocytes were prepared from the vaccinated mice and stimulated with either HPV16 E6 (aa 50 to 57), HPV16 E7 (aa 49 to 57), or HPV18 E6 (aa 67 to 75) peptide in the presence of GolgiPlug. Intracellular IFN-γ cytokine staining assay was performed to detect antigen-specific CD8^+^ T cells. The cells were acquired with a FACSCalibur flow cytometer, and data were analyzed with CellQuest Pro software. (B) Summary of the HPV16 E7 tetramer flow cytometry analysis. (C) Intracellular IFN-γ cytokine staining assay after stimulation with either HPV16 E6 (aa 50 to 57) peptide (5 μg/ml), HPV16 E7 (aa 49 to 57) peptide (1 μg/ml), or HPV18 E6 (aa 67 to 75) peptide (1 μg/ml).

The use of recombinant vaccinia, such as TA-HPV, is known to enhance both the T cell response and antitumor immunity elicited after DNA vaccination; however, without a priming vaccination, it is poorly effective (22, 31). Naive 6- to 8-week-old female C57BL/6 mice (5 mice/group) were vaccinated with pBI-11 DNA (25 μg/50 μl/mouse) through i.m. injections. The mice were boosted with the same regimen 7 days later. One week after the second vaccination, one group of the mice was again vaccinated with pBI-11 DNA for sequential vaccination with the pBI-11 DNA for a total of three times (DDD regimen), whereas another group of mice was vaccinated with TA-HPV (1 × 10^6^ PFU/50 μl/mouse) by i.m. injection and thus were vaccinated with pBI-11 twice followed by TA-HPV vaccination once (DDV regimen). PBMCs were collected 6 days after the last vaccination and characterized for HPV16 E7-specific CD8^+^ T cell-mediated immune responses using HPV16 E7 peptide-loaded tetramer staining followed by flow cytometry analysis (Fig. 6A), and splenocytes were collected 2 weeks after final treatment (Fig. 6A). Mice vaccinated with the DDV regimen had significantly higher percentages of E7-specific CD8^+^ T cells than mice vaccinated with pBI-11 alone (DDD) (*P* = 0.0100) (Fig. 6B). We also characterized HPV16 E7- and HPV18 E6-specific CD8^+^ T cell-mediated immune responses using splenocytes from vaccinated mice (Fig. 6C). Mice in a DNA prime-vaccinia boost (DDV) had significantly higher numbers of HPV16 E7-specific T cells (*P* = 0.0428) and higher, although not significantly so, HPV18 E6-specific T cells (*P* = 0.2116) than those that only received DNA vaccination (Fig. 6C). We previously characterized HPV16 E7-specific CD8^+^ T cell-mediated immune responses in mice vaccinated with TA-HPV alone (22). We found that mice vaccinated with TA-HPV alone did not generate appreciable HPV16 E7-specific CD8^+^ T cell-mediated immune responses (22). Thus, TA-HPV booster vaccination is capable of simultaneously enhancing HPV16 and HPV18 antigen-specific CD8^+^ T cell immune responses generated after priming with pBI-11 DNA vaccine.

### pBI-11 DNA prime TA-HPV vaccinia boost regimen can be combined with anti-PD-1 immune checkpoint blockade to improve therapeutic antitumor response.

**FIG 7:**
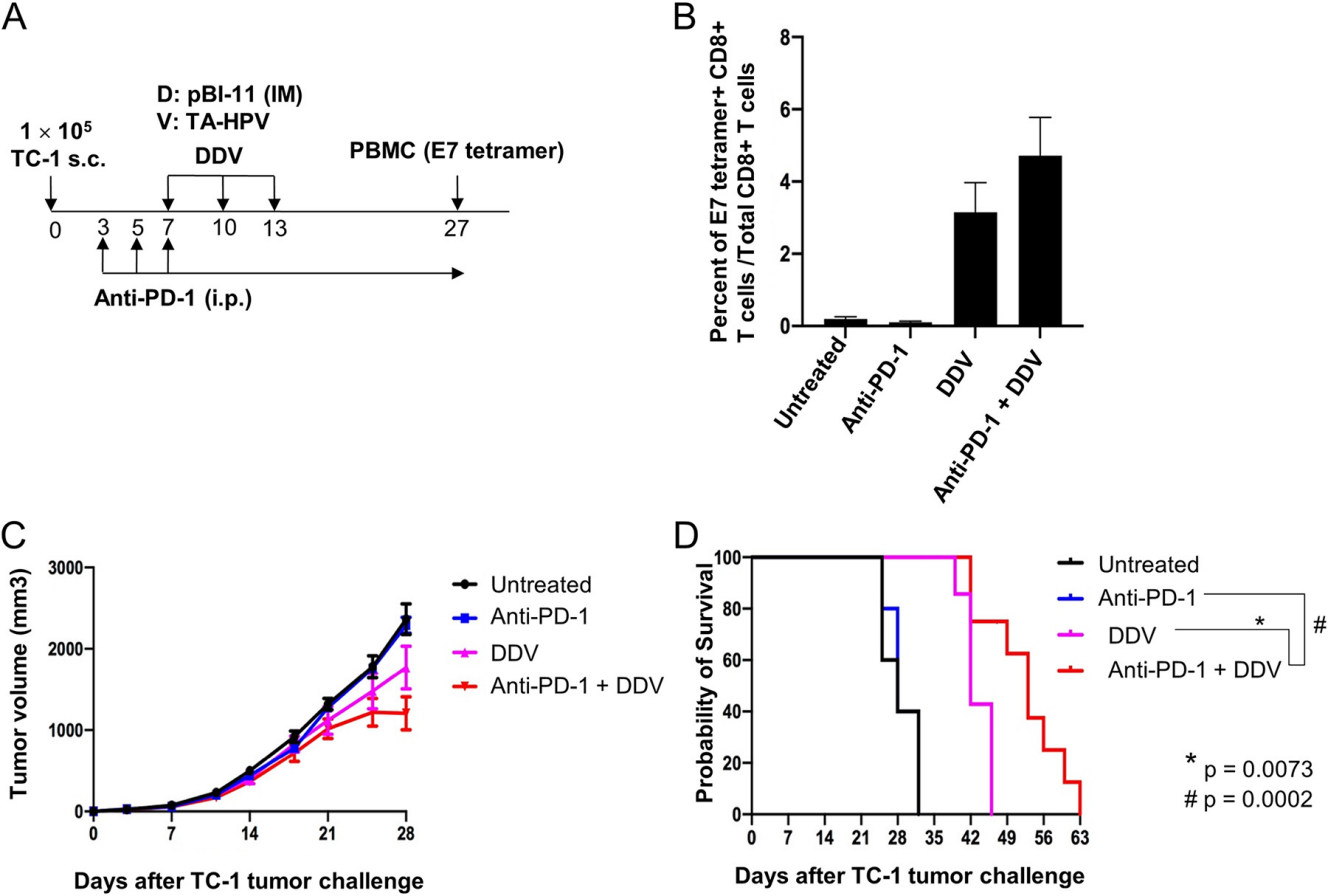
Characterization of the HPV antigen-specific immune response and antitumor effects in a TC-1 tumor-bearing mouse treated with DDV with or without anti-PD-1. Six- to eight-week-old female C57BL/6 mice (5 to 8 mice/group) were injected with 2 × 10^5^ of TC-1 cells subcutaneously on day 0. On day 3, the mice were divided into 4 groups. The first group was used as the untreated control. The second group was injected with purified anti-mouse PD-1 monoclonal antibody (MAb; clone 29F.1A12, 200 μg/mouse) via intraperitoneal injection. The treatment was repeated every other day. The third group was vaccinated with pBI-11 DNA (25 μg/50 μl/mouse) through i.m. injection and boosted once 3 days later. The mice were further boosted with TA-HPV vaccinia virus 3 days later through skin scarification. The fourth group was treated with both anti-mouse PD-1 MAb and pBI-11 DNA vaccine prime followed by TA-HPV vaccinia virus boost as described in Materials and Methods. (A) Schema of the experiment. (B) Detection of HPV16 E7-specific CD8^+^ T cells in peripheral blood using HPV16 E7 tetramer staining. (C) Summary of the TC-1 tumor volumes in the mice. (D) Kaplan-Meier analysis of the survival of TC-1 tumor-bearing mice.

PD-1 antibody blockade is used to treat patients with recurrent/refractory cervical cancer. Therefore, we examined the safety and efficacy of combining the pBI-11 DNA and TA-HPV vaccine regimen with the PD-1 antibody. Six- to eight-week-old female C57BL/6 mice (5 to 8 mice/group) were injected with 2 × 10^5^ TC-1 cells subcutaneously on day 0. On day 3, the mice were divided into 4 groups. The first group was used as the untreated control. The second group was administered anti-mouse PD-1 monoclonal antibody (MAb; 200 μg/mouse) via intraperitoneal injection on the regimen indicated above. The third group was vaccinated i.m. with pBI-11 DNA (25 μg/50 μl/mouse) and boosted once 3 days later. The mice were further boosted with TA-HPV vaccinia virus 3 days later through skin scarification (DDV). The fourth group was treated with both anti-mouse PD-1 MAb and pBI-11 DNA vaccine prime followed by TA-HPV vaccinia virus boost as described above (DDV) (Fig. 7A). On day 27, PBMCs were collected for the characterization of HPV16 E7-specific CD8^+^ T cell-mediated immune responses using HPV16 E7 peptide (aa 49 to 57)-loaded tetramer staining. As shown in Fig. 7B, mice receiving DDV vaccination, either alone or with anti-PD-1 antibody, displayed E7-specific CD8^+^ T cell-mediated immune responses, whereas in the absence of vaccination with anti-PD-1 antibody, treatment did not elicit a detectable HPV16 E7-specific CD8^+^ T cell response (Fig. 7B). Furthermore, addition of anti-PD-1 antibody treatment to the DDV regimen significantly enhanced the therapeutic antitumor effects, although it had no discernible effect alone (Fig. 7C). This suggests synergy of vaccination and anti-PD-1 antibody treatment and that the latter is not effective without a prior immune response. Furthermore, the combinational treatment (anti-PD-1 plus DDV) translated into significantly (*P* = 0.0073 when compared to DDV, and *P* = 0.0002 when compared to anti-PD-1) better survival of the tumor-bearing mice (Fig. 7D).

### pBI-11 DNA vaccination and two pBI-11 priming vaccinations followed by a single recombinant vaccinia virus vaccine TA-HPV boost is well tolerated.

Clinical trials with TA-HPV have suggested the vaccine is well tolerated (17, 20, 21). Similarly, heterologous prime-boost vaccination trials with pBI-1 DNA and TA-HPV have been well tolerated in humans (19). However, it is unclear whether the new pBI-11 DNA construct alone or the combination of pBI-11 DNA with TA-HPV will be similarly well tolerated. To address this issue, 6- to 8-week-old female naive C57BL/6 mice (5 mice per group) were divided into 3 groups. The mice in the first group were vaccinated with phosphate-buffered saline (PBS) (50 μl/mouse) via i.m. injection in the left hind leg, and the mice were boosted twice with the same regimen at 1-week intervals (PBS). The mice of the second group were vaccinated with pBI-11 DNA (25 μg/50 μl/mouse) through i.m. injection in the left hind leg and boosted twice with the same regimen at 1-week intervals (DDD). The mice in the third group were vaccinated with pBI-11 DNA (25 μg/50 μl/mouse) through i.m. injection and boosted once with the same regimen at a 1-week interval. One week after the second pBI-11 DNA administration, these mice were further boosted once with 1 × 10^6^ PFU (50 μl/mouse) of TA-HPV through i.m. injection in the left hind leg (DDV) (see Fig. S1 in the supplemental material). All materials used were clinical grade.

10.1128/mBio.03224-20.1 FIG S1 Schematic illustration of toxicology study experimental design. Briefly, mice were administered vaccine doses on days 0, 7, and 14. Edema and eschar formation were monitored on days of vaccination and up to 24 h postvaccination. Behavior and body weight were assessed at all timepoints. One week after final vaccination, splenocytes were collected, and organs and blood were collected for histology, complete blood counts, and biochemistry analysis. Download FIG S1, TIF file, 0.05 MB. Copyright © 2021 Peng et al. 2021 Peng et al. This content is distributed under the terms of the Creative Commons Attribution 4.0 International license.

We first compared HPV antigen-specific CD8^+^ T cell-mediated immune responses between DDV, DDD, and PBS control vaccination regimens. Although we completed flow cytometry 1 week after final vaccination rather than after 2 weeks (Fig. 5C), the overall trend of DDV-vaccinated mice generating a higher immune response to HPV16 E7 and HPV18 E6 antigenic peptides than DDD-vaccinated mice remained, and successful vaccination was demonstrated (see Fig. S2). The health of the mice was monitored by the measurement of behaviors, body weight, and injection site irritation throughout the duration of vaccination and up to 1 week post-final vaccination (Fig. S1). All vaccinated mice appeared healthy during the whole experimental period. Mice in all three treatment groups demonstrated typical healthy behavioral phenotypes for the duration of the experiment (see Table S1). Similarly, the body weights of the mice in all vaccination groups remained comparable throughout the duration of the experiment (Fig. S3). In general, there was a slight increase in mouse weight toward the end of the study, and this shift was observed in all vaccination groups without a significant difference. On the days that mice were vaccinated, they were observed for injection site reaction 2 and 24 h postvaccination. DDV and DDD vaccinations did not cause eschar (Table S2) or edema (Table S3) formation at any time point, similar to that for the PBS control.

10.1128/mBio.03224-20.2 FIG S2 Characterization of the HPV antigen-specific CD8^+^ T cell-mediated immune response by intracellular cytokine stain for IFN-γ followed by flow cytometry analysis. Seven days after the last vaccination, splenocytes were prepared and stimulated with either HPV16 E6 (aa 50 to 57) peptide (5 μg/ml), or HPV16 E7 (aa 49 to 57) peptide (1 μg/ml), or HPV18 E6 (aa 67 to 75) peptide (1 μg/ml) in the presence of GolgiPlug (1 μl/ml) overnight. The cells were stained with PE-conjugated anti-mouse CD8a (clone 53.6.7, 1 μl/sample) at 4°C for 30 min. After washing, the cells were permeabilized and fixed with perm/fix buffer (from eBioscience) at 4°C for 30 min. After washing, the cells were further stained with FITC-conjugated anti-mouse IFN-γ (clone XMG1.2, 1 μl/sample) at 4°C for 45 min. After washing, the cells were resuspended in PBS plus 0.5% BSA. The cells were acquired with a FACSCalibur flow cytometer, and data were analyzed with CellQuest Pro software. The bar graph is representative of immune response in C57BL/6 mice (*n*= 5) vaccinated with the PBS, DDD, or DDV vaccination regimen. One week after the final vaccination, splenocytes were prepared and stimulated with either HPV16 E6 (aa 50 to 57) peptide (5 μg/ml), HPV16 E7 (aa 49 to 57) peptide (1 μg/ml), or HPV18 E6 (aa 67 to 75) peptide (1 μg/ml) in the presence of GolgiPlug (1 μl/ml) overnight. In HPV16 E7 aa (49 to 57) peptide-specific CD8^+^ T cells, DDD versus DDV has a *P* value of 0.0234. In HPV18 E6 (aa 67 to 75) peptide-specific CD8^+^ T cells, DDV versus DDV has a *P* value of 0.0216. Data shown represent means ± standard deviations (SDs). Download FIG S2, TIF file, 0.1 MB. Copyright © 2021 Peng et al. 2021 Peng et al. This content is distributed under the terms of the Creative Commons Attribution 4.0 International license.

10.1128/mBio.03224-20.3 FIG S3 Characterization of body weight following the initial vaccination over time. Represented as the mean weight of each of the vaccinated C57BL/6 mice in DDV, DDD, and PBS groups. *N* = 5. 0, day of initial vaccination. Body weights of the mice were measured at indicated times with a digital scale (model CS200; Ohaus Corp., USA,). No significant difference in mean body weights among different vaccinated groups. Data shown represent means ± SDs at each time point. Download FIG S3, TIF file, 0.08 MB. Copyright © 2021 Peng et al. 2021 Peng et al. This content is distributed under the terms of the Creative Commons Attribution 4.0 International license.

10.1128/mBio.03224-20.5 TABLE S1 Behavioral phenotypes of mice receiving PBS, DDD, or DDV vaccination treatment. The mice received vaccine injections on 29 September 2020, 6 October 2020, and 13 October 2020 with PBS, DDD or DDV. Mice were sacrificed October 2020. N (no) and 2 represent normal behaviors. Numerical rating scale 0 to 3. Download Table S1, DOCX file, 0.02 MB. Copyright © 2021 Peng et al. 2021 Peng et al. This content is distributed under the terms of the Creative Commons Attribution 4.0 International license.

10.1128/mBio.03224-20.6 TABLE S2 Assessment for eschar formation at the vaccinated site. WNL/NSF indicates within normal limits/no significant findings (unremarkable). Download Table S2, DOCX file, 0.02 MB. Copyright © 2021 Peng et al. 2021 Peng et al. This content is distributed under the terms of the Creative Commons Attribution 4.0 International license.

10.1128/mBio.03224-20.7 TABLE S3 Assessment for edema formation at the vaccinated site. WNL/NSF indicates within normal limits/no significant findings (unremarkable). Download Table S3, DOCX file, 0.02 MB. Copyright © 2021 Peng et al. 2021 Peng et al. This content is distributed under the terms of the Creative Commons Attribution 4.0 International license.

Necropsy was performed 1 week after the last vaccination, and the key organ weight measurement, complete blood count, clinical chemistry analysis, and histological studies were performed. The mean organ weights were comparable in PBS, DDD, and DDV vaccination groups (Fig. S4), and a portion of the spleen of each mouse was used for analysis of response to vaccination (Fig. S1). Complete blood count measurements were generally unremarkable and similar between vaccination groups (Table S4). While not all biochemistry readouts were available for all mouse subjects, available biochemistry results demonstrated that PBS, DDD, and DDV vaccination groups were similar (Table S5). The histopathology analysis of the major organs revealed no significant findings, which were similar between the PBS, DDD, and DDV vaccination groups (Table S6). Taken together, the similar behavioral and physiological status of mice from the two active treatment groups versus that of the control mice vaccinated with buffer only suggests that priming with pBI-11 DNA vaccine followed by boosting with TA-HPV vaccinia vaccine is safe and well tolerated in mice as is a repeat pBI-11 DNA vaccination regimen at a 1-week interval.

10.1128/mBio.03224-20.4 FIG S4 Characterization of key organ weights 1 week after final vaccination. Represented as mean organ weights of each of the vaccinated C57BL/6 mice in DDV, DDD, and PBS groups. *N *= 5. Seven days after the last vaccination, the mice were sacrificed, and heart (A), lung with trachea (B), liver (C), spleen (D), left kidney (E), and right kidney (F) were collected, and the weights were measured with a digital scale (model AB104-S; Mettler Toledo, Columbus, OH, USA). No significant difference in mean organ weights among different vaccinated groups. Data shown represent means ± SDs. Download FIG S4, TIF file, 0.1 MB. Copyright © 2021 Peng et al. 2021 Peng et al. This content is distributed under the terms of the Creative Commons Attribution 4.0 International license.

10.1128/mBio.03224-20.8 TABLE S4 Complete blood count of vaccinated mice. Summary of complete blood count studies in vaccinated mice using serum 1 week after final vaccination. RBC, red blood cell count; HGB, hemoglobin value; HCT, hematocrit value; MCV, mean corpuscular volume; MHC, mean corpuscular hemoglobin; MCHC, mean corpuscular hemoglobin concentration; RDW-SD, red cell distribution width standard deviation; RDW-CV, red cell distribution width coefficient of variation; RET, reticulocytes; IRF, immature reticulocyte fraction; LFR, low fluorescence ratio; MFR, medium fluorescence ratio; HFR, high fluorescence ratio; RET-He, retic hemoglobin; PLT, platelet count; PDW, platelet distribution width; MPV, mean platelet volume; P-LCR, platelet/large cell ratio; PCT, plateletcrit value; WBC, white blood cell count; NEUT, neutrophil percent; LYMPH, lymphocyte percent; MONO, monocyte; EO, eosinophil; BASO, basophil. Download Table S4, DOCX file, 0.02 MB. Copyright © 2021 Peng et al. 2021 Peng et al. This content is distributed under the terms of the Creative Commons Attribution 4.0 International license.

10.1128/mBio.03224-20.9 TABLE S5 Summary of the biochemistry study in vaccinated mice using serum 1 week after final vaccination. ALB, albumin; ALB/GLOB, albumin to globulin; ALP, alkaline phosphatase; ALT, alanine aminotransferase; AMY, amylase; AST, aspartate aminotransferase; BUN, blood urea nitrogen; BUN/CREA, blood urea nitrogen to creatinine; CA, calcium; CA (ALB), calcium corrected for albumin; CA (TP), calcium corrected for total protein; PHOS, phosphate; CHOL, cholesterol; CK, creatine kinase; CL, chloride; CREA, creatinine; GGT, gamma-glutamyl transferase; GLUC, glucose; K, potassium; LDH, lactic acid dehydrogenase; MG, magnesium; NA, sodium; TBIL, total bilirubin; TP, total protein; TRIG, triglyceride; UA, uric acid; – , data not available due to machine reading error and limited serum. Download Table S5, DOCX file, 0.02 MB. Copyright © 2021 Peng et al. 2021 Peng et al. This content is distributed under the terms of the Creative Commons Attribution 4.0 International license.

10.1128/mBio.03224-20.10 TABLE S6 Histological examination of key organs in mice receiving vaccination. Organs were examined after the mice were sacrificed on 20 October 2020, 1 week after final vaccination. WNL/NSF, within normal limits/no significant findings (unremarkable). NT, no tissue. Download Table S6, DOCX file, 0.02 MB. Copyright © 2021 Peng et al. 2021 Peng et al. This content is distributed under the terms of the Creative Commons Attribution 4.0 International license.

## DISCUSSION

Here, we have described the construction and characterization of several therapeutic HPV DNA vaccines that encode the E6/E7 oncogenic proteins of HPV16 and HPV18 as a secreted fusion protein fused with the alarmin M. tuberculosis HSP70 to enhance the induction of cellular immune response after administration (Fig. 1). There are multiple strategies to improve therapeutic potential of HPV vaccinations beyond the employment of Sig and HSP70 protein strategy (for review, see reference 40). For example, ubiquitin has been linked to the encoded HPV antigen in the context of naked DNA vaccines to improve the antigen processing and presentation and through MHC class I molecules to improve DNA vaccine potency (41). In addition, Igk, a typical leader (signal) sequence, has been constructed in the mammalian expression vector containing the cytomegalovirus promoter to enhance the secretion of the linked protein expressed in transfected cells to improve DNA vaccine potency (42). In comparison, the HSP70 strategy used in our DNA vaccine targets and concentrates the secreted HPV fusion protein linked to the professional antigen-presenting cells to improve cross-presentation of the HPV antigens linked to HSP70. Thus, these strategies employ different molecules linked to the antigen to enhance DNA vaccine potency through different mechanisms.

We continued to characterize one of these codon-optimized DNA vaccines, pBI-11, alone and in combination with vaccinia virus vaccine TA-HPV and a PD-1 immune checkpoint blockade to enhance HPV antigen-specific CD8^+^ T cell-mediated immune responses and the control of HPV16^+^ tumors. The focus on maximizing HPV early protein antigen-specific CD8 T cell response differs from that of preventative HPV vaccines that aim to produce neutralizing antibodies, as the latter have no impact on infected cells. As HPV-associated cancer cells obligately express only early proteins E6 and E7, cytotoxic T lymphocytes against these early proteins are most relevant, and immune escape is less likely.

In the present studies, we did not observe a significant HPV16 E6-specific CD8^+^ T cell-mediated immune response in the vaccinated mice. This effect may be attributed to cytotoxic T lymphocyte (CTL) immunodominance, as strong HPV16 E7-specific and/or HPV18 E6-specific responses may overwhelm the response to the weak HPV16 E6-specific epitope. This hypothesis is corroborated by a study in which vaccination with a DNA vaccine encoding HPV16 E6 antigen alone was capable of generating appreciable HPV16 E6-specific CD8^+^ T cell-mediated immune responses when in the absence of HPV16 E7 antigen (39). These data are consistent with previous reports that the presence of an HPV16 E7-specific H-2D^b^-restricted CTL epitope can preclude the presentation of HPV16 E6-specific HLA-H2-restricted CTL epitopes, demonstrating that immunodominant CTL epitopes are able to suppress other nonimmunodominant epitopes (43).

It is important to consider using multiple antigens in vaccine development. Using more HPV antigens for the development of therapeutic HPV vaccines would make them efficacious in a genetically diverse population, as not all human MHC class I molecules can present a specific HPV antigen with similar efficacy. For example, we previously tested vaccination with a DNA vaccine encoding HPV16 E7 antigen in different human MHC class I transgenic mice, and we only observed a significant HPV16 E7-specific CD8^+^ T cell-mediated immune response in the vaccinated HLA-A2 transgenic mice. In comparison, HLA-A1, HPA-A11, HLA-A24, HLA-B7, and HLA-B44 all demonstrated markedly lower immune responses to the vaccination with the DNA vaccine (data not shown). Therefore, it is critical to incorporate multiple HPV antigens in vaccine development so that a broadly diverse human population may generate an appreciable HPV antigen-specific CD8^+^ T cell-mediated immune response.

Several concerns have to be addressed before the clinical-grade pBI-11 can be moved to the clinics. One concern is the oncogenicity of HPV E6/E7. In the present studies, we mutated several key amino acids in E6/E7 of HPV16/18 to eliminate the oncogenic potential of pBI-11. Another concern is the potential to induce autoimmunity by vaccinating with these novel sequences. Thus, we have searched for the potential expression of novel peptides derived from HPV16/18 E6/E7 proteins identical to endogenous self-peptides. We did not find any peptides composed of more than seven amino acids derived from the E6/E7 of HPV16/18 identical to endogenous self-peptides. Since most of the CTL epitopes are around 8 to 11 amino acids, it is unlikely that the vaccination with pBI-11 will generate HPV antigen-specific CTL activities that will cross-react to endogenous protein/peptides. Furthermore, vaccination strategies using different vaccines, including TA-HPV, derived from the HPV16/18 E6/E7 antigens have been used in several clinical trials with acceptable safety profiles, and no serious side effects related to autoimmune diseases have been reported (17, 18, 20, 21, 44, 45).

Here, we have demonstrated that priming with pBI-11 DNA followed by boosting with TA-HPV in conjunction with anti-PD-1 can generate significant antitumor effects (Fig. 7). The FDA has approved pembrolizumab (KEYTRUDA) for use in cervical cancer patients, and several other ongoing clinical trials are investigating the safety and efficacy of other checkpoint-blocking antibodies in cervical cancer (for review, see reference 34). In general, immune checkpoint-blocking antibodies have demonstrated some degree of efficacy against cervical cancer and HPV-associated head and neck cancers (35, 46). Elevated levels of PD-L1/PD-1 tend to be associated with better clinical responses to immune checkpoint blockade. We have previously shown that therapeutic vaccination with an HPV16 L2E7E6 fusion protein, TA-CIN, in the TC-1 tumor model upregulated PD-L1 on tumor cells and PD-1 on circulating CD8^+^ T cells (47). Other groups have also reported that HPV-associated cancers tend to be associated with upregulated PD-L1 and enhanced responses to immune checkpoint blockades (35, 48–50). Furthermore, PD-1/PD-L1 blockade has been used in conjunction with other cancer vaccines to improve therapeutic effects (51, 52). Thus, the upregulation of the PD-1/PD-L1 axis post-therapeutic vaccination may be mitigated by use of immune checkpoint blockades, therefore improving overall antitumor effect. Here, we showed that a heterologous vaccine strategy of pBI-11 and TA-HPV combined with anti-PD-1 antibody elicited a strong antitumor response, leading to a better survival than with vaccination alone (Fig. 7).

pBI-1 has already been used in multiple clinical trials and was shown to be well tolerated (19). pBI-1 is the first-generation version of pBI-11: pBI-11 targets HPV16 and HPV18 E6/E7, whereas pBI-1 targets only HPV16 E7. Additionally, TA-HPV has demonstrated safety, tolerability, and immunogenicity in several clinical trials. Furthermore, pBI-1 DNA vaccination followed by a single TA-HPV vaccinia boost was well tolerated (19). Based on these previous clinical trials and the absence of observed side effects in the preclinical studies herein (see Tables S1 to S6 in the supplemental material), pBI-11 is expected to be safe for use in patients either alone or with TA-HPV. Importantly, compared to pBI-1, pBI-11 has the added benefit of targeting three more HPV antigens and an additional HPV type, HPV18. Thus, pBI-11 may better serve as the priming DNA vaccine for TA-HPV to generate multiple HPV antigen-specific CD8^+^ T cell-mediated immune system responses to control the two most oncogenic hrHPV infections and HPV-associated diseases in a genetically diverse population. In addition, it may also induce cross-reactive therapeutic T cell responses against closely related hrHPV types in the alpha 7 and alpha 9 genera (53). In summary, the observed safety profile and therapeutic efficacy of pBI-11 in preclinical studies alone and when used in conjunction with TA-HPV, with or without PD-1 antibody blockade therapy, suggest that early-phase clinical testing is warranted.

## MATERIALS AND METHODS

### Design and synthesis of candidate HPV DNA vaccine constructs.

The pBI-1 DNA vaccine has been described previously as pNGVL4a-SigE7(detox)HSP70 (28). The pBI-10.1, pBI-11, and pBI-12 DNA constructs were derived by Gibson assembly of a DNA fragment synthesized by Bio Basic (Markham, ON, Canada) encoding a fusion protein of the signal peptide, HPV16 E7 (detox), HPV18 E7 (detox), HPV16 E6 (detox), and HPV18 E6 (detox), arranged in an order different from that in TA-HPV to avoid boosting junction-associated epitopes, as well as a 5′ portion of HSP70 (up to the Tth111I site) flanked 5′ by an EcoRI and Kozak site and 3′ with a Tth111I site. The sequences were either codon optimized for gene expression in human cells using Bio Basic’s algorithm (Fig. 1A, red boxes) or were based on native papillomaviral sequences (Fig. 1A, blue boxes). The synthesized fragment was cloned into the pBI-1 to replace the fragment between EcoRI and Tth111I (1011 and 1480) in frame with HSP70. The pBI-10.1, pBI-11, and pBI-12 DNA constructs were manufactured and validated by restriction digestions and DNA sequencing. The pBI-11 DNA construct map is shown in Fig. 1B

In pBI-1, pBI-10.1, pBI-11, and pBI-12, the HPV16 E7(detox) (19, 30, 54) contains mutations C24G and E26G that eliminate E7’s transforming function and binding to the retinoblastoma protein (pRB) (55, 56). In addition, pBI-10.1, pBI-11, and pBI-12 carry a C91G mutation to destroy E7’s single zinc finger in conserved region 3 (CR3) (57), which alone eliminates the immortalizing activity of E7 as well as binding to histone deacetylase (HDAC), c-jun, and BRCA1 (58–61). The stop codon is removed from E7 to permit fusion with HPV18 E7. A parallel set of inactivating mutations have also been introduced into HPV18 E7(detox), specifically, C27G (62), E29G (16), and C98G (63), and the stop codon was removed from HPV18 E7 to permit fusion with HPV16 E6. The HPV16 E6(detox) gene contains multiple mutations to disrupt oncogenic activity by targeting key cysteine residues in the two zinc finger domains via C63G and C106G mutations. This approach was previously used in a vaccine construct and shown to eliminate the ability to trigger the degradation of p53 (57). In another study, mutation of either cysteine residue was shown to abolish the immortalization activity of HPV16 E6 (64). Likewise, cells transduced with E6 containing mutations of either C63G or C106G retained normal levels of p53, whereas p53 was almost undetectable in cells expressing wild-type E6 (64). The C63G mutation also serves to knock out the activation of telomerase by E6 (65). To further eliminate potential HPV16 E6 activity from its second zinc finger motif, the C-terminal 5 residues containing the PDZ domain were also deleted (66–69), and the stop codon was deleted for direct fusion to HPV18 E6. A parallel set of inactivating mutations have also been introduced into HPV18 E6 (detox): C65G and C108G (70–73). To further eliminate potential HPV18 E6 activity from its second zinc finger motif, the C-terminal 5 residues containing the PDZ domain were also deleted (66), and the stop codon was deleted for direct fusion to HSP70, as in pBI-1.

### Analysis of potential for expression of novel peptides derived from HPV16/18 E6/E7 proteins identical to endogenous self-peptides.

A potential concern from the addition of HPV18 E7(detox) and HPV16 and HPV18 E6(detox) to pBI-1 is that they could encode peptides with sequences common to host proteins that induce cross-reactive immunity against self-antigens. In preclinical studies, vaccination with pBI-1 DNA induced robust CD8 T cell immunity without measurable antibody responses (24, 32). Likewise, in a phase I clinical study, vaccination with pBI-1 elicited an E7-specific CD8 T cell response but no detectable antibody response (30). Therefore, we sought to determine the likelihood of inducing an autoimmune T cell response in patients. To identify vaccine epitopes that might induce cross-reactivity against self-antigens, we first compared the sequences of vaccine peptides to those of human proteins. Considering that peptide antigens are presented as fragments of either 8 to 11 amino acids on MHC class I to CD8 T cells, or 12 to 20 amino acids on MHC class II to CD4 T cells (for review, see reference 74), we carried out a search for all 8-mers generated from the HPV16/18 E6/E7 peptides plus the junctional regions in the pBI-11-encoded fusion protein against human protein sequences in UniProt (75), which contains the Swiss-Prot and TrEMBL databases. To search for potential novel peptides that may be identical to endogenous peptides, we generated all linear sequences of 5 to 8 amino acids in length (5-mers to 8-mers) from amino acid 24 to 550 of pBI-11, which span the last 7 amino acids of the signal peptide, HPV16 E7(detox), HPV18 E7(detox), HPV18 E6(detox), HPV16 E6(detox), and the first 11 amino acids of HSP70 (Fig. 1). In total, 520 8-mers, 521 7-mers, 522 6-mers, and 523 5-mers were generated. We submitted the sequences to the UniProt protein database (https://www.uniprot.org/) in groups of 80 to 100 sequences using the Peptide Search Tool provided at the website and searched for exact matches against the Swiss-Prot and TrEMBL databases filtered for human proteins. Search results revealed no exact match between vaccine and endogenous human peptide sequences that are at least 8 amino acids in length. For comparison, a search for all 7-mers which are below the minimum size for a T cell epitope identified 16 entries in Swiss-Prot and 88 entries in TrEMBL, corresponding to 10 unique sequences mapped to 12 unique endogenous human proteins (Table 1), whereas searches for 6-mers and 5-mers returned >4,000 and 2 million entries, respectively. However, these sequence identities of ≤7 amino acids are too small for MHC presentation.

**TABLE 1:** 7-mers from pBI-11 protein mapped to human proteins in the UniProt database

HPV protein	Peptide	Human protein	UniProt ID
HPV18 E7	ELVVESS	Vacuolar protein sorting-associated protein 26C (Down syndrome critical region protein 3) (Down syndrome critical region protein A) (VPS26C, DCRA, DSCR3, DSCRA)	O14972
	VNHQHLP	T-cell differentiation antigen CD6 (T12) (TP120) (CD antigen CD6)	P30203
	LPARRAE	Pre-mRNA-splicing factor SYF1 (protein HCNP) (XPA-binding protein 2) (XAB2, HCNP, KIAA1177, SYF1, PP3898)	Q9HCS7
	SDSEEEN	Laminin subunit beta-4 (laminin beta-1-related protein) (LAMP4)	A4D0S4
	LSDSEEE	Exosome complex component RRP45 (autoantigen PM/Scl 1) (exosome component 9) (P75 polymyositis-scleroderma overlap syndrome-associated autoantigen) (polymyositis/scleroderma autoantigen 1) (polymyositis/scleroderma autoantigen 75 kDa) (PM/Scl-75) (EXOSC9, PMSCL1)	Q06265
	DSEEEND	DDB1- and CUL4-associated factor 11 (DCAF11, WDR23, GL014, PRO2389)	Q8TEB1
HPV18 E6	KPLCDLL	Importin subunit alpha-5, -6, -7 (KPNA1, KPNA5, KPNA6)	P52294 O15131 O60684
HPV16 E6	FEFAFKD	DNA-directed RNA polymerase I subunit RPA2 (RNA polymerase I subunit 2) (EC 2.7.7.6) (DNA-directed RNA polymerase I 135-kDa polypeptide) (RPA135) (POLR1B)	Q9H9Y6
	TVLELTE	Plasma protease C1 inhibitor (C1 Inh) (C1Inh) (C1 esterase inhibitor) (C1-inhibiting factor) (Serpin G1) (SERPING1, C1IN, C1NH)	P05155
	RARQERL	Caldesmon (CALD1, CAD, CDM)	Q05682

As an alternative approach, we also searched the Immune Epitope Database (https://www.iedb.org/) (76) using the same region of the pBI-11 protein sequence. We selected “substring” for linear epitope to obtain any epitope sequences that are mapped to the pBI-10.1 sequence. Our search identified a B cell epitope derived from HPV16 E7 (RTLED; glutamate decarboxylase 2 [GAD2], A0A3B3IU09). This epitope is also present in pBI-1, which has been tested for its safety in human subjects (19, 30). Taken together, these analyses indicate that the addition of HPV18 E7(detox) and HPV16 and HPV18 E6(detox) to pBI-1 is unlikely to generate peptides that could induce cross-reactive T cell immunity against self-antigens.

### Mice.

Six- to eight-week-old female C57BL/6 mice were purchased from Taconic Biosciences (Germantown, NY). All mice were housed ≤5/cage in 12-h light/12-h dark at 68 to 79°F and 30% to 70% humidity with both purified water and Envigo Teklad certified rodent chow 2018C *ad libitum* in the animal facility under specific-pathogen-free conditions at Johns Hopkins University School of Medicine (Baltimore, MD). Animals were quarantined 1 week prior to use and individually identified by a unique ear punch or by marker pen. All procedures were performed according to preapproved protocols and in accordance with recommendations for the proper use and care of laboratory animals.

### Peptides, antibodies, and other reagents.

HPV16 E6 (aa 50 to 57) peptide, YDFAFRDL, HPV16 E7 (aa 49 to 57) peptide, RAHYNIVTF, and HPV18 E6 (aa 67 to 75) peptide, KCIDFYSRI, were synthesized by GenScript (Piscataway, NJ) at a purity of ≥80%. Fluorescein isothiocyanate (FITC) and phycoerythrin (PE)-conjugated anti-mouse CD8a (clone 53.6.7), FITC-conjugated anti-mouse gamma interferon (IFN-γ) (clone XMG1.2) antibodies, and 7-aminoactinomycin D (7-AAD) were purchased from BioLegend (San Diego, CA). Purified anti-mouse PD-1 monoclonal antibody (clone 29F.1A12) and purified rat anti-mouse CD16/32 (clone 2.4G2) were purchased from Bio X Cell (West Lebanon, NH). PE-conjugated HPV16 E7 (aa 49 to 57) peptide-loaded H-2D^b^ tetramers and PE-conjugated HPV18 E6 (aa 67 to 75) peptide-loaded H-2K^b^ tetramers were purchased from MBL International (Japan). Bovine serum albumin (BSA) was purchased from Sigma (St. Louis, MO). Lipofectamine 2000 was purchased from Invitrogen (Waltham, MA). Opti-MEM I medium was purchased from Gibco (Gaithersburg, MD). Recombinant murine interleukin 2 (IL-2) was purchased from R&D Systems (Minneapolis, MN). Bifurcated needles were purchased from Precision Medical Products, Inc. (Denver, PA).

### Cells.

HPV16 E6- and E7-expressing TC-1 cells were generated as previously described (77). The cells were maintained in RPMI medium supplemented with 2 mM glutamine, 1 mM sodium pyruvate, 100 IU ml^−1^ penicillin, 100 μg ml^−1^ streptomycin, and 10% fetal bovine serum (FBS). HEK 293 expi cells were acquired from ATCC (Manassas, VA). The generation of 293 cells expressing the murine MHC class I molecule D^b^, 293-D^b^, or K^b^, 293-K^b^, was described previously (78). 293 expi, 293-Db, and 293-K^b^ cells were cultured in Dulbecco’s modified Eagle medium (DMEM) containing 2 mM glutamine, 1 mM sodium pyruvate, 100 IU ml^−1^ penicillin, 100 μg ml^−1^ streptomycin, and 10% FBS. The establishment of the murine HPV16 E7 peptide (aa 49 to 57)-specific CD8^+^ T cell line was described previously (79). These T cells were restimulated with irradiated TC-1 cells weekly in the presence of recombinant murine IL-2. To establish murine HPV18 E6 (aa 67 to 75) peptide-specific CD8^+^ T cells, 6- to 8-week-old naive female C57BL/6 mice were vaccinated with a DNA vaccine, pCDNA3-CRT/18E6 (38), three times through intramuscular injection followed by electroporation. Splenocytes were harvested 7 days after the last vaccination and stimulated with irradiated HPV18 E6 (aa 67 to 75) peptide-loaded TC-1 cells in the presence of recombinant murine IL-2 and restimulated weekly.

### Western blot analysis.

Western blotting was performed for E7 and E6 in 293 expi cells transfected with pBI-1, pBI-10.1.1, pBI-11, or pBI-12. To identify the protein expression level of HPV16-E7 and HPV16/18-E6/E7 fusion proteins, 293 expi cells were transfected with 10 μg of each plasmid. At 48 h after transfection, total cell lysates were collected for Western blot analysis. Equivalent amounts of total cell lysates were separated on a precast Tris-HCl protein gel (Life Technology, Rockville, MD, USA) and then transferred onto a nitrocellulose membrane (Bio-Rad). After blocking, the membrane was hybridized with anti-HPV16 E7 monoclonal antibody (8C9 clone from Invitrogen), anti-HPV18/16 E6 (CIP5 clone from Abcam), or anti-GAPDH (catalog number [no.] 60004-1-Ig; Proteintech). Antibody binding was detected using a peroxidase-conjugated sheep anti-mouse secondary antibody (Amersham, Piscataway, NJ, USA) and chemiluminescence (ECL^+^ detection kit; Amersham). The density of the band correlating to the HPV16/18 E6 fusion protein expressed was measured by densitometry with a Bio-Rad ChemiDoc imaging system and analyzed by Bio-Rad Image Lab software.

### *In vitro* HPV16 E7 and HPV18 E6 antigen presentation assay.

To compare the capacity for HPV16 E7 and HPV18 E6 antigen presentation by cells transfected with each of the DNA vaccine constructs, 293-D^b^ or 293-K^b^ cells were transfected with either pBI-10.1, pBI-11, or pBI-12 DNA vaccine or mock transfected using Lipofectamine 2000. Twenty-four hours later, transfected 293-D^b^ cells were harvested, and 2.5 × 10^5^ cells were cocultured with 2.5 × 10^5^ HPV16 E7 peptide (aa 49 to 57)-specific CD8^+^ T cells per well in a 96-well round-bottom plate in the presence of GolgiPlug (BD Pharmingen, San Diego, CA) for 20 to 24 h. Likewise, 24 h after transfection, transfected 293-K^b^ cells were harvested and cocultured with HPV18 E6 peptide (aa 67 to 75)-specific CD8^+^ T cells in the presence of GolgiPlug. The cells were then harvested and stained for intracellular IFN-γ. Flow cytometry analysis was performed to detect the activation of HPV-16 E7 or HPV 18 E6 peptide-specific CD8^+^ T cells.

### Vaccination.

TA-HPV is a recombinant vaccinia virus expressing HPV16/18-E6/E7, and its preparation has been described previously (18). TA-HPV has been used in several clinical trials including patients with cervical cancer (17, 18), cervical intraepithelial neoplasia (17, 80), vulvar intraepithelial neoplasia (20), vaginal intraepithelial neoplasia (21), and noncervical anogenital intraepithelial neoplasias (21, 44), and it was prepared and vialed under cGMP by Omnia Biomanufacturing, Rockville, MD, at 10^7^ PFU/ml (lot no. 0054-16007). DNA was prepared using Qiagen Endofree kits, except as indicated for pBI-11 when it was prepared and vialed under cGMP by Waisman Biomanufacturing, Madison, WI, at 3 mg/ml in PBS (lot no. PPV-pBI-11-FP-001). DNA was administered in PBS to C57BL/6 mice via intramuscular (i.m.) injections in the hind legs. Animals were vaccinated with TA-HPV by either i.m. injection or by skin scarification. For TA-HPV skin scarification, mice were anesthetized, and 5 μl of the vaccine at the designated dose was applied to tail skin 1 cm from the base of the tail or on the ear. The skin area was then gently scratched 15 times with a bifurcated needle (Precision Medical Products, Inc., Denver, PA). When vaccination schedules required a booster vaccination, the contralateral leg was used for vaccination, and subsequent vaccinations were performed alternating the hind legs.

### Tetramer staining.

For tetramer staining, mouse PBMCs were stained with purified anti-mouse CD16/32 first and then stained with FITC-conjugated anti-mouse CD8a and PE-conjugated HPV16/E7 (aa 49 to 57) peptide-loaded H-2D^b^ tetramer or PE-conjugated HPV18 E6 (aa 67 to 75) peptide-loaded H-2K^b^ tetramer at 4°C for 1 h. After washing, the cells were stained with 7-AAD. The cells were acquired with the FACSCalibur flow cytometer and analyzed with CellQuest Pro software (BD Biosciences, Mountain View, CA).

### Intracellular cytokine staining and flow cytometry analysis.

To detect HPV16 E6-, E7-, or HPV18 E6-specific CD8^+^ T cell responses by IFN-γ intracellular staining, splenocytes were stimulated with either HPV 16 E6 (aa 50 to 57) peptide (5 μg/ml), HPV16 E7 (aa 49 to 57) peptide (1 μg/ml), or HPV18 E6 (aa 67 to 75) peptide (1 μg/ml) in the presence of GolgiPlug (1 μl/ml, 1:1,000 dilution of the antibody; BD Pharmingen, San Diego, CA) at 37°C overnight. The stimulated splenocytes were then washed with PBS containing 0.5% BSA and stained with PE-conjugated anti-mouse CD8a (1 μl/sample, 1:1,000 dilution of the antibody) at 4°C for 30 min. After washing, the cells were fixed and permeabilized using the Cytofix/Cytoperm kit according to the manufacturer’s instructions (eBioscience, San Diego, CA) at 4°C for 30 min. After washing, intracellular IFN-γ was stained with FITC-conjugated anti-mouse IFN-γ (1 μl/sample) at 4°C for 45 min. After washing, the cells were resuspended in PBS plus 0.5% BSA. Flow cytometry analysis was performed using a FACSCalibur flow cytometer with CellQuest software.

### *In vivo* tumor treatment experiment.

For the *in vivo* tumor treatment experiment, 6- to 8-week-old female C57BL/6 mice (five per group) were implanted with 2 × 10^5^ of TC-1 tumor cells subcutaneously. The tumor-bearing mice were vaccinated as indicated in Results and treated with purified anti-mouse PD-1 antibody (clone 29F.1A12; Bio X Cell, West Lebanon, NH) at the dose of 200 μg/mouse via intraperitoneal injection every other day. The growth of the tumor was monitored twice a week by palpation and digital caliper measurement. Tumor volume was calculated using the formula [largest diameter × (perpendicular diameter)^2^] × 3.14/6. To record the survival of the tumor-bearing mice, either natural death or a tumor diameter greater than 2 cm leading to death was counted as death.

### Assessment for impact of vaccination on behavior and physiological status of mice.

Female naive C57BL/6 mice (5 mice per group) were vaccinated twice with clinical-grade pBI-11 DNA (lot no. PPV-pBI11-FP-001; Waisman Biomanufacturing, Madison, WI) at a dose of 25 μg in 50 μl/mouse and then once with clinical grade TA-HPV (lot no. 0054-16007; Omnia Biologics Inc., Rockville, MD) at a dose of 1 × 10^6^ PFU/50 μl/mouse (DDV), three times with only clinical-grade PBI-11 DNA (DDD), or three times with only PBS (as a control) at 1-week intervals on days 0, 7, and 14 (see Fig. S1 in the supplemental material). All the injections were performed at the same location in the hind legs. Each vaccine was administered to the mice via intramuscular injection. The health of the mice was monitored by the measurement of behaviors, body weight, and injection site irritation throughout the duration of vaccination and up to 1-week post-final vaccination (Fig. S1) per the JHU Animal Pathobiology and Phenotyping manual. In addition, necropsy was performed 1 week after the last vaccination (day 21), key organ weights were measured, and histology was examined by a board-certified pathologist (32). Approximately one-half of each spleen was used for histologic analysis, and the remainder was used to prepare single splenocytes and stimulated with HPV16 E6/E7 and HPV18 E6 peptide followed by IFN-γ intracellular staining.

### Statistical analysis.

Data are summarized by descriptive statistics, including means and standard deviations. Individual data points were compared by Student’s *t* tests. Survival functions for mice in different groups were estimated by the Kaplan-Meier estimator and compared by the log rank test. No multiplicity control was considered because of the exploratory nature of the analyses. A *P* value of less than 0.05 was considered significant. Statistical analysis was performed using Prism 8 software (GraphPad).
