# Supplementary figures and images for: Development of DNA Vaccine Targeting E6 and E7 Proteins of Human Papillomavirus 16 (HPV16) and HPV18 for Immunotherapy in Combination with Recombinant Vaccinia Boost and PD-1 Antibody
Source: mBio. 2021 Jan 19;12(1):e03224-20. doi: 10.1128/mBio.03224-20 (PMC7845631; doi:10.1128/mBio.03224-20)

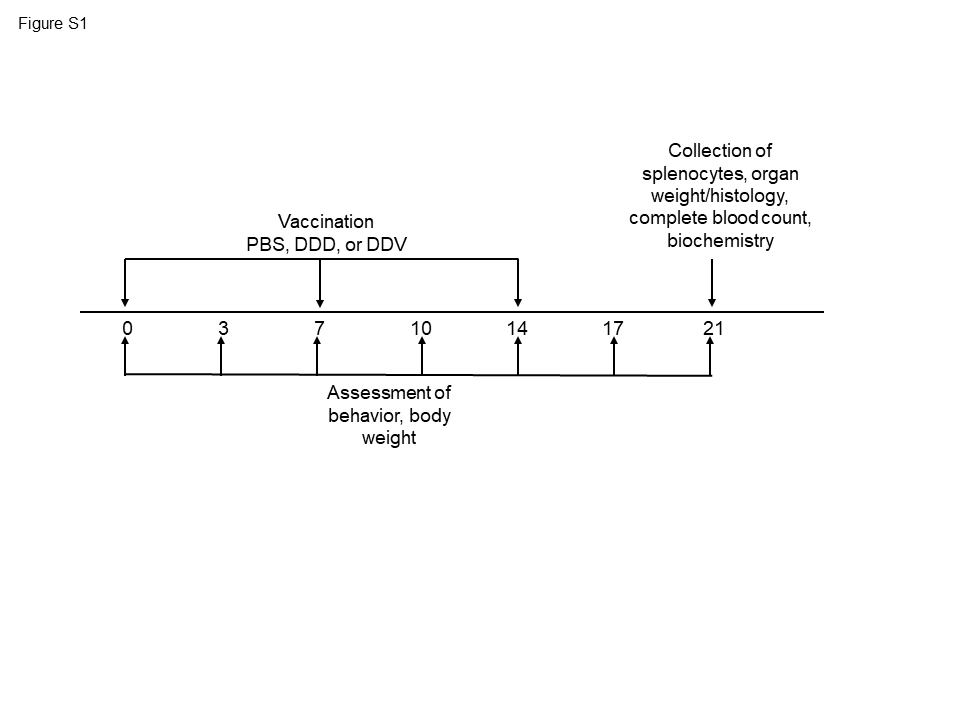

Supplement: FIG S1 [file mBio.03224-20-sf001.tif]

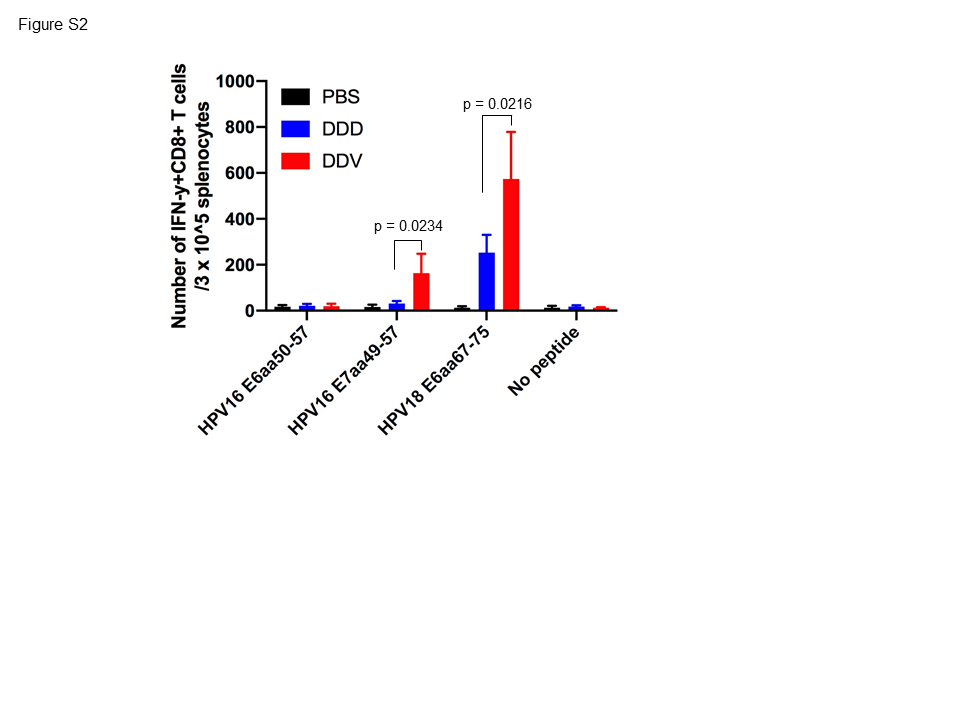

Supplement: FIG S2 [file mBio.03224-20-sf002.tif]

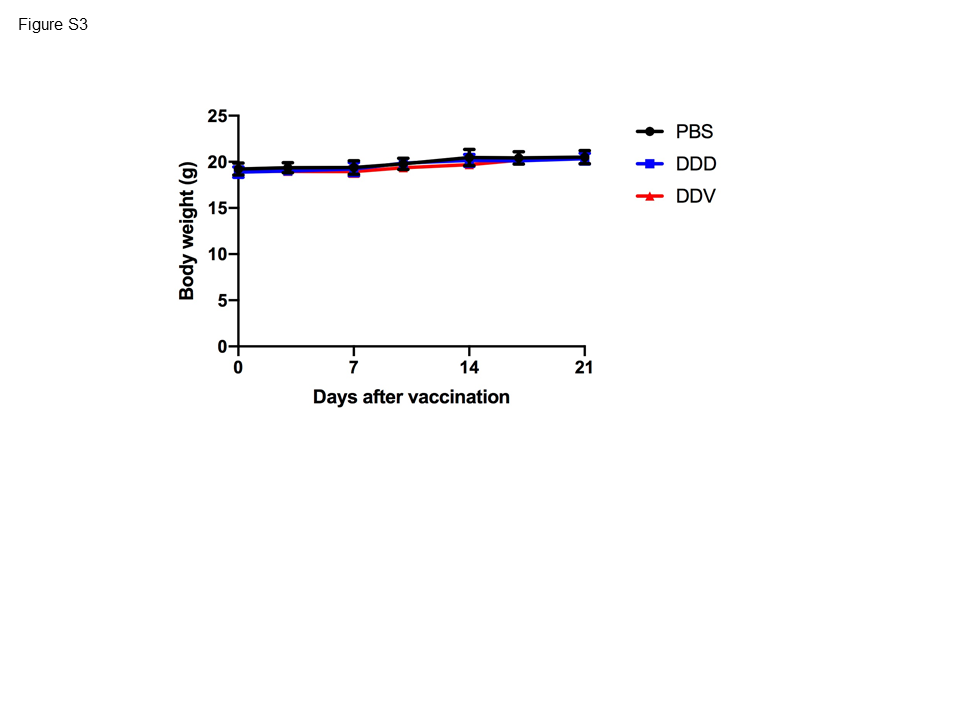

Supplement: FIG S3 [file mBio.03224-20-sf003.tif]

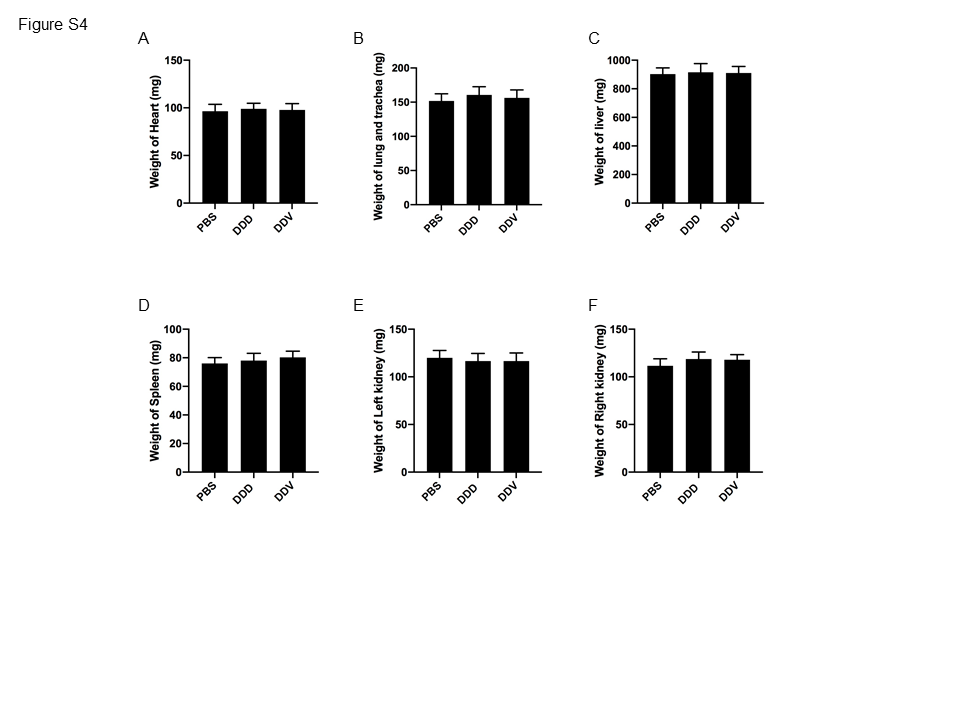

Supplement: FIG S4 [file mBio.03224-20-sf004.tif]
