# Supplementary material for: Development of DNA Vaccine Targeting E6 and E7 Proteins of Human Papillomavirus 16 (HPV16) and HPV18 for Immunotherapy in Combination with Recombinant Vaccinia Boost and PD-1 Antibody
Source: mBio. 2021 Jan 19;12(1):e03224-20. doi: 10.1128/mBio.03224-20 (PMC7845631; doi:10.1128/mBio.03224-20)
Supplement: TABLE S1 [file mBio.03224-20-st001.docx]

**Table S1**: .Behavioral Phenotype of Mice Receiving PBS, DDD, or DDV vaccination treatment. The mice received vaccine injections at 09/29/2020, 10/6/2020 and 10/13/2020 with PBS, DDD or DDV. Mice were sacrificed 10/20/20/20. N (no) and 2 represent normal behaviors. Numerical rating scale 0-3.

Assessment of Abnormal Gait in vaccinated mice

| Vaccination Group | Vaccination Mouse Number | Observation Date 9/29/20 | Observation Date 10/2/20 | Observation Date 10/6/20 | Observation Date 10/9/20 | Observation Date 10/13/20 | Observation Date 10/16/20 | Observation Date 10/20/20 |
| --- | --- | --- | --- | --- | --- | --- | --- | --- |
| PBS | PBS-1 | N | N | N | N | N | N | N |
|  | PBS-2 | N | N | N | N | N | N | N |
|  | PBS-3 | N | N | N | N | N | N | N |
|  | PBS-4 | N | N | N | N | N | N | N |
|  | PBS-5 | N | N | N | N | N | N | N |
| DDD | DDD-1 | N | N | N | N | N | N | N |
|  | DDD-2 | N | N | N | N | N | N | N |
|  | DDD-3 | N | N | N | N | N | N | N |
|  | DDD-4 | N | N | N | N | N | N | N |
|  | DDD-5 | N | N | N | N | N | N | N |
| DDV | DDV-1 | N | N | N | N | N | N | N |
|  | DDV-2 | N | N | N | N | N | N | N |
|  | DDV-3 | N | N | N | N | N | N | N |
|  | DDV-4 | N | N | N | N | N | N | N |
|  | DDV-5 | N | N | N | N | N | N | N |

N represents typical behavior.

Assessment of Abnormal Posture in vaccinated mice

| Vaccination Group | Vaccination Mouse Number | Observation Date 9/29/20 | Observation Date 10/2/20 | Observation Date 10/6/20 | Observation Date 10/9/20 | Observation Date 10/13/20 | Observation Date 10/16/20 | Observation Date 10/20/20 |
| --- | --- | --- | --- | --- | --- | --- | --- | --- |
| PBS | PBS-1 | N | N | N | N | N | N | N |
|  | PBS-2 | N | N | N | N | N | N | N |
|  | PBS-3 | N | N | N | N | N | N | N |
|  | PBS-4 | N | N | N | N | N | N | N |
|  | PBS-5 | N | N | N | N | N | N | N |
| DDD | DDD-1 | N | N | N | N | N | N | N |
|  | DDD-2 | N | N | N | N | N | N | N |
|  | DDD-3 | N | N | N | N | N | N | N |
|  | DDD-4 | N | N | N | N | N | N | N |
|  | DDD-5 | N | N | N | N | N | N | N |
| DDV | DDV-1 | N | N | N | N | N | N | N |
|  | DDV-2 | N | N | N | N | N | N | N |
|  | DDV-3 | N | N | N | N | N | N | N |
|  | DDV-4 | N | N | N | N | N | N | N |
|  | DDV-5 | N | N | N | N | N | N | N |

N represents typical behavior.

Assessment of Freezing Behavior

| Vaccination Group | Vaccination Mouse Number | Observation Date 9/29/20 | Observation Date 10/2/20 | Observation Date 10/6/20 | Observation Date 10/9/20 | Observation Date 10/13/20 | Observation Date 10/16/20 | Observation Date 10/20/20 |
| --- | --- | --- | --- | --- | --- | --- | --- | --- |
| PBS | PBS-1 | N | N | N | N | N | N | N |
|  | PBS-2 | N | N | N | N | N | N | N |
|  | PBS-3 | N | N | N | N | N | N | N |
|  | PBS-4 | N | N | N | N | N | N | N |
|  | PBS-5 | N | N | N | N | N | N | N |
| DDD | DDD-1 | N | N | N | N | N | N | N |
|  | DDD-2 | N | N | N | N | N | N | N |
|  | DDD-3 | N | N | N | N | N | N | N |
|  | DDD-4 | N | N | N | N | N | N | N |
|  | DDD-5 | N | N | N | N | N | N | N |
| DDV | DDV-1 | N | N | N | N | N | N | N |
|  | DDV-2 | N | N | N | N | N | N | N |
|  | DDV-3 | N | N | N | N | N | N | N |
|  | DDV-4 | N | N | N | N | N | N | N |
|  | DDV-5 | N | N | N | N | N | N | N |

N represents typical behavior.

Wild Running

| Vaccination Group | Vaccination Mouse Number | Observation Date 9/29/20 | Observation Date 10/2/20 | Observation Date 10/6/20 | Observation Date 10/9/20 | Observation Date 10/13/20 | Observation Date 10/16/20 | Observation Date 10/20/20 |
| --- | --- | --- | --- | --- | --- | --- | --- | --- |
| PBS | PBS-1 | N | N | N | N | N | N | N |
|  | PBS-2 | N | N | N | N | N | N | N |
|  | PBS-3 | N | N | N | N | N | N | N |
|  | PBS-4 | N | N | N | N | N | N | N |
|  | PBS-5 | N | N | N | N | N | N | N |
| DDD | DDD-1 | N | N | N | N | N | N | N |
|  | DDD-2 | N | N | N | N | N | N | N |
|  | DDD-3 | N | N | N | N | N | N | N |
|  | DDD-4 | N | N | N | N | N | N | N |
|  | DDD-5 | N | N | N | N | N | N | N |
| DDV | DDV-1 | N | N | N | N | N | N | N |
|  | DDV-2 | N | N | N | N | N | N | N |
|  | DDV-3 | N | N | N | N | N | N | N |
|  | DDV-4 | N | N | N | N | N | N | N |
|  | DDV-5 | N | N | N | N | N | N | N |

N represents typical behavior.

Stereotypies

| Vaccination Group | Vaccination Mouse Number | Observation Date 9/29/20 | Observation Date 10/2/20 | Observation Date 10/6/20 | Observation Date 10/9/20 | Observation Date 10/13/20 | Observation Date 10/16/20 | Observation Date 10/20/20 |
| --- | --- | --- | --- | --- | --- | --- | --- | --- |
| PBS | PBS-1 | N | N | N | N | N | N | N |
|  | PBS-2 | N | N | N | N | N | N | N |
|  | PBS-3 | N | N | N | N | N | N | N |
|  | PBS-4 | N | N | N | N | N | N | N |
|  | PBS-5 | N | N | N | N | N | N | N |
| DDD | DDD-1 | N | N | N | N | N | N | N |
|  | DDD-2 | N | N | N | N | N | N | N |
|  | DDD-3 | N | N | N | N | N | N | N |
|  | DDD-4 | N | N | N | N | N | N | N |
|  | DDD-5 | N | N | N | N | N | N | N |
| DDV | DDV-1 | N | N | N | N | N | N | N |
|  | DDV-2 | N | N | N | N | N | N | N |
|  | DDV-3 | N | N | N | N | N | N | N |
|  | DDV-4 | N | N | N | N | N | N | N |
|  | DDV-5 | N | N | N | N | N | N | N |

N represents typical behavior.

Escape

| Vaccination Group | Vaccination Mouse Number | Observation Date 9/29/20 | Observation Date 10/2/20 | Observation Date 10/6/20 | Observation Date 10/9/20 | Observation Date 10/13/20 | Observation Date 10/16/20 | Observation Date 10/20/20 |
| --- | --- | --- | --- | --- | --- | --- | --- | --- |
| PBS | PBS-1 | N | N | N | N | N | N | N |
|  | PBS-2 | N | N | N | N | N | N | N |
|  | PBS-3 | N | N | N | N | N | N | N |
|  | PBS-4 | N | N | N | N | N | N | N |
|  | PBS-5 | N | N | N | N | N | N | N |
| DDD | DDD-1 | N | N | N | N | N | N | N |
|  | DDD-2 | N | N | N | N | N | N | N |
|  | DDD-3 | N | N | N | N | N | N | N |
|  | DDD-4 | N | N | N | N | N | N | N |
|  | DDD-5 | N | N | N | N | N | N | N |
| DDV | DDV-1 | N | N | N | N | N | N | N |
|  | DDV-2 | N | N | N | N | N | N | N |
|  | DDV-3 | N | N | N | N | N | N | N |
|  | DDV-4 | N | N | N | N | N | N | N |
|  | DDV-5 | N | N | N | N | N | N | N |

N represents typical behavior.

Exploring

| Vaccination Group | Vaccination Mouse Number | Observation Date 9/29/20 | Observation Date 10/2/20 | Observation Date 10/6/20 | Observation Date 10/9/20 | Observation Date 10/13/20 | Observation Date 10/16/20 | Observation Date 10/20/20 |
| --- | --- | --- | --- | --- | --- | --- | --- | --- |
| PBS | PBS-1 | 2 | 2 | 2 | 2 | 2 | 2 | 2 |
|  | PBS-2 | 2 | 2 | 2 | 2 | 2 | 2 | 2 |
|  | PBS-3 | 2 | 2 | 2 | 2 | 2 | 2 | 2 |
|  | PBS-4 | 2 | 2 | 2 | 2 | 2 | 2 | 2 |
|  | PBS-5 | 2 | 2 | 2 | 2 | 2 | 2 | 2 |
| DDD | DDD-1 | 2 | 2 | 2 | 2 | 2 | 2 | 2 |
|  | DDD-2 | 2 | 2 | 2 | 2 | 2 | 2 | 2 |
|  | DDD-3 | 2 | 2 | 2 | 2 | 2 | 2 | 2 |
|  | DDD-4 | 2 | 2 | 2 | 2 | 2 | 2 | 2 |
|  | DDD-5 | 2 | 2 | 2 | 2 | 2 | 2 | 2 |
| DDV | DDV-1 | 2 | 2 | 2 | 2 | 2 | 2 | 2 |
|  | DDV-2 | 2 | 2 | 2 | 2 | 2 | 2 | 2 |
|  | DDV-3 | 2 | 2 | 2 | 2 | 2 | 2 | 2 |
|  | DDV-4 | 2 | 2 | 2 | 2 | 2 | 2 | 2 |
|  | DDV-5 | 2 | 2 | 2 | 2 | 2 | 2 | 2 |

2 represents normal behavior.

Digging

| Vaccination Group | Vaccination Mouse Number | Observation Date 9/29/20 | Observation Date 10/2/20 | Observation Date 10/6/20 | Observation Date 10/9/20 | Observation Date 10/13/20 | Observation Date 10/16/20 | Observation Date 10/20/20 |
| --- | --- | --- | --- | --- | --- | --- | --- | --- |
| PBS | PBS-1 | 2 | 2 | 2 | 2 | 2 | 2 | 2 |
|  | PBS-2 | 2 | 2 | 2 | 2 | 2 | 2 | 2 |
|  | PBS-3 | 2 | 2 | 2 | 2 | 2 | 2 | 2 |
|  | PBS-4 | 2 | 2 | 2 | 2 | 2 | 2 | 2 |
|  | PBS-5 | 2 | 2 | 2 | 2 | 2 | 2 | 2 |
| DDD | DDD-1 | 2 | 2 | 2 | 2 | 2 | 2 | 2 |
|  | DDD-2 | 2 | 2 | 2 | 2 | 2 | 2 | 2 |
|  | DDD-3 | 2 | 2 | 2 | 2 | 2 | 2 | 2 |
|  | DDD-4 | 2 | 2 | 2 | 2 | 2 | 2 | 2 |
|  | DDD-5 | 2 | 2 | 2 | 2 | 2 | 2 | 2 |
| DDV | DDV-1 | 2 | 2 | 2 | 2 | 2 | 2 | 2 |
|  | DDV-2 | 2 | 2 | 2 | 2 | 2 | 2 | 2 |
|  | DDV-3 | 2 | 2 | 2 | 2 | 2 | 2 | 2 |
|  | DDV-4 | 2 | 2 | 2 | 2 | 2 | 2 | 2 |
|  | DDV-5 | 2 | 2 | 2 | 2 | 2 | 2 | 2 |

2 represents normal behavior.

Grooming

| Vaccination Group | Vaccination Mouse Number | Observation Date 9/29/20 | Observation Date 10/2/20 | Observation Date 10/6/20 | Observation Date 10/9/20 | Observation Date 10/13/20 | Observation Date 10/16/20 | Observation Date 10/20/20 |
| --- | --- | --- | --- | --- | --- | --- | --- | --- |
| PBS | PBS-1 | 2 | 2 | 2 | 2 | 2 | 2 | 2 |
|  | PBS-2 | 2 | 2 | 2 | 2 | 2 | 2 | 2 |
|  | PBS-3 | 2 | 2 | 2 | 2 | 2 | 2 | 2 |
|  | PBS-4 | 2 | 2 | 2 | 2 | 2 | 2 | 2 |
|  | PBS-5 | 2 | 2 | 2 | 2 | 2 | 2 | 2 |
| DDD | DDD-1 | 2 | 2 | 2 | 2 | 2 | 2 | 2 |
|  | DDD-2 | 2 | 2 | 2 | 2 | 2 | 2 | 2 |
|  | DDD-3 | 2 | 2 | 2 | 2 | 2 | 2 | 2 |
|  | DDD-4 | 2 | 2 | 2 | 2 | 2 | 2 | 2 |
|  | DDD-5 | 2 | 2 | 2 | 2 | 2 | 2 | 2 |
| DDV | DDV-1 | 2 | 2 | 2 | 2 | 2 | 2 | 2 |
|  | DDV-2 | 2 | 2 | 2 | 2 | 2 | 2 | 2 |
|  | DDV-3 | 2 | 2 | 2 | 2 | 2 | 2 | 2 |
|  | DDV-4 | 2 | 2 | 2 | 2 | 2 | 2 | 2 |
|  | DDV-5 | 2 | 2 | 2 | 2 | 2 | 2 | 2 |

2 represents normal behavior.

Rearing

| Vaccination Group | Vaccination Mouse Number | Observation Date 9/29/20 | Observation Date 10/2/20 | Observation Date 10/6/20 | Observation Date 10/9/20 | Observation Date 10/13/20 | Observation Date 10/16/20 | Observation Date 10/20/20 |
| --- | --- | --- | --- | --- | --- | --- | --- | --- |
| PBS | PBS-1 | 2 | 2 | 2 | 2 | 2 | 2 | 2 |
|  | PBS-2 | 2 | 2 | 2 | 2 | 2 | 2 | 2 |
|  | PBS-3 | 2 | 2 | 2 | 2 | 2 | 2 | 2 |
|  | PBS-4 | 2 | 2 | 2 | 2 | 2 | 2 | 2 |
|  | PBS-5 | 2 | 2 | 2 | 2 | 2 | 2 | 2 |
| DDD | DDD-1 | 2 | 2 | 2 | 2 | 2 | 2 | 2 |
|  | DDD-2 | 2 | 2 | 2 | 2 | 2 | 2 | 2 |
|  | DDD-3 | 2 | 2 | 2 | 2 | 2 | 2 | 2 |
|  | DDD-4 | 2 | 2 | 2 | 2 | 2 | 2 | 2 |
|  | DDD-5 | 2 | 2 | 2 | 2 | 2 | 2 | 2 |
| DDV | DDV-1 | 2 | 2 | 2 | 2 | 2 | 2 | 2 |
|  | DDV-2 | 2 | 2 | 2 | 2 | 2 | 2 | 2 |
|  | DDV-3 | 2 | 2 | 2 | 2 | 2 | 2 | 2 |
|  | DDV-4 | 2 | 2 | 2 | 2 | 2 | 2 | 2 |
|  | DDV-5 | 2 | 2 | 2 | 2 | 2 | 2 | 2 |

2 represents normal behavior.
