# Supplementary material for: Development of DNA Vaccine Targeting E6 and E7 Proteins of Human Papillomavirus 16 (HPV16) and HPV18 for Immunotherapy in Combination with Recombinant Vaccinia Boost and PD-1 Antibody
Source: mBio. 2021 Jan 19;12(1):e03224-20. doi: 10.1128/mBio.03224-20 (PMC7845631; doi:10.1128/mBio.03224-20)
Supplement: TABLE S3 [file mBio.03224-20-st003.docx]

**Table S3**: Assessment of edema formation at vaccinated site

|  |  | **Vaccination Date (9/29/2020)** | | **Vaccination Date (10/6/2020)** | | **Vaccination Date (10/13/2020)** | |
| --- | --- | --- | --- | --- | --- | --- | --- |
| Vaccination Group | Vaccination Mouse Number | 2 hours | 24 hours | 2 hours | 24 hours | 2 hours | 24 hours |
| **PBS** | PBS-1 | WNL/NSF | WNL/NSF | WNL/NSF | WNL/NSF | WNL/NSF | WNL/NSF |
|  | PBS-2 | WNL/NSF | WNL/NSF | WNL/NSF | WNL/NSF | WNL/NSF | WNL/NSF |
|  | PBS-3 | WNL/NSF | WNL/NSF | WNL/NSF | WNL/NSF | WNL/NSF | WNL/NSF |
|  | PBS-4 | WNL/NSF | WNL/NSF | WNL/NSF | WNL/NSF | WNL/NSF | WNL/NSF |
|  | PBS-5 | WNL/NSF | WNL/NSF | WNL/NSF | WNL/NSF | WNL/NSF | WNL/NSF |
| **DDD** | DDD-1 | WNL/NSF | WNL/NSF | WNL/NSF | WNL/NSF | WNL/NSF | WNL/NSF |
|  | DDD-2 | WNL/NSF | WNL/NSF | WNL/NSF | WNL/NSF | WNL/NSF | WNL/NSF |
|  | DDD-3 | WNL/NSF | WNL/NSF | WNL/NSF | WNL/NSF | WNL/NSF | WNL/NSF |
|  | DDD-4 | WNL/NSF | WNL/NSF | WNL/NSF | WNL/NSF | WNL/NSF | WNL/NSF |
|  | DDD-5 | WNL/NSF | WNL/NSF | WNL/NSF | WNL/NSF | WNL/NSF | WNL/NSF |
| **DDV** | DDV-1 | WNL/NSF | WNL/NSF | WNL/NSF | WNL/NSF | WNL/NSF | WNL/NSF |
|  | DDV-2 | WNL/NSF | WNL/NSF | WNL/NSF | WNL/NSF | WNL/NSF | WNL/NSF |
|  | DDV-3 | WNL/NSF | WNL/NSF | WNL/NSF | WNL/NSF | WNL/NSF | WNL/NSF |
|  | DDV-4 | WNL/NSF | WNL/NSF | WNL/NSF | WNL/NSF | WNL/NSF | WNL/NSF |
|  | DDV-5 | WNL/NSF | WNL/NSF | WNL/NSF | WNL/NSF | WNL/NSF | WNL/NSF |

WNL/NSF – Within normal limits / No significant findings (unremarkable)
