# Supplementary material for: Development of DNA Vaccine Targeting E6 and E7 Proteins of Human Papillomavirus 16 (HPV16) and HPV18 for Immunotherapy in Combination with Recombinant Vaccinia Boost and PD-1 Antibody
Source: mBio. 2021 Jan 19;12(1):e03224-20. doi: 10.1128/mBio.03224-20 (PMC7845631; doi:10.1128/mBio.03224-20)
Supplement: TABLE S4 [file mBio.03224-20-st004.docx]

**Table S4**: Complete blood count of vaccinated mice.

| Mouse Number | 1 | 2 | 3 | 4 | 5 | 6 | 7 | 8 | 9 | 10 | 11 | 12 | 13 | 14 | 15 |
| --- | --- | --- | --- | --- | --- | --- | --- | --- | --- | --- | --- | --- | --- | --- | --- |
| Test Day | 10/20/2020 | 10/20/2020 | 10/20/2020 | 10/20/2020 | 10/20/2020 | 10/20/2020 | 10/20/2020 | 10/20/2020 | 10/20/2020 | 10/20/2020 | 10/20/2020 | 10/20/2020 | 10/20/2020 | 10/20/2020 | 10/20/2020 |
| Species | Mouse | Mouse | Mouse | Mouse | Mouse | Mouse | Mouse | Mouse | Mouse | Mouse | Mouse | Mouse | Mouse | Mouse | Mouse |
| Strain | C57BL/6 | C57BL/6 | C57BL/6 | C57BL/6 | C57BL/6 | C57BL/6 | C57BL/6 | C57BL/6 | C57BL/6 | C57BL/6 | C57BL/6 | C57BL/6 | C57BL/6 | C57BL/6 | C57BL/6 |
| Age (week) | 20 | 20 | 20 | 20 | 20 | 20 | 20 | 20 | 20 | 20 | 20 | 20 | 20 | 20 | 20 |
| Sex | Female | Female | Female | Female | Female | Female | Female | Female | Female | Female | Female | Female | Female | Female | Female |
| Vaccination Group Number | DDV-1 | DDV-2 | DDV-3 | DDV-4 | DDV-5 | DDD-1 | DDD-2 | DDD-3 | DDD-4 | DDD-5 | PBS-1 | PBS-2 | PBS-3 | PBS-4 | PBS-5 |
| RBC (M/uL) | 10.80 | 10.66 | 10.80 | 10.48 | 11.24 | 10.35 | 10.65 | 10.93 | 11.01 | 10.64 | 11.04 | 10.57 | 10.62 | 10.97 | 11.11 |
| HGB (g/dL) | 16.7 | 16.2 | 16.4 | 15.8 | 17.0 | 15.6 | 16.3 | 16.4 | 16.6 | 16.2 | 16.9 | 16.0 | 16.3 | 16.7 | 16.8 |
| HCT (%) | 49.6 | 49.5 | 49.7 | 47.6 | 52.0 | 47.8 | 50.1 | 51.6 | 51.3 | 49.9 | 51.9 | 49.6 | 49.8 | 52.0 | 51.9 |
| MCV (fL) | 45.9 | 46.4 | 46.0 | 45.4 | 46.3 | 46.2 | 47.0 | 47.2 | 46.6 | 46.9 | 47.0 | 46.9 | 46.9 | 47.4 | 46.7 |
| MCH (pg) | 15.5 | 15.2 | 15.2 | 15.1 | 15.1 | 15.1 | 15.3 | 15.0 | 15.1 | 15.2 | 15.3 | 15.1 | 15.3 | 15.2 | 15.1 |
| MCHC (g/dL) | 33.7 | 32.7 | 33.0 | 33.2 | 32.7 | 32.6 | 32.5 | 31.8 | 32.4 | 32.5 | 32.6 | 32.3 | 32.7 | 32.1 | 32.4 |
| RDW-SD (fL) | 27.6 | 27.5 | 27.3 | 26.9 | 27.7 | 27.4 | 26.9 | 26.5 | 26.5 | 26.9 | 26.8 | 26.7 | 27.2 | 27.0 | 26.5 |
| RDW-CV (%) | 24.7 | 24.4 | 24.5 | 24.2 | 25.1 | 24.1 | 23.9 | 23.7 | 23.8 | 23.7 | 24.0 | 23.7 | 23.6 | 24.1 | 23.9 |
| RET (K/ul) | 399.6 | 379.5 | 381.2 | 318.6 | 388.9 | 331.2 | 345.1 | 337.7 | 366.6 | 383.0 | 338.9 | 303.4 | 367.5 | 427.8 | 307.7 |
| IRF (%) | 56.2 | 59.6 | 66.8 | 56.1 | 59.6 | 60.5 | 58.0 | 56.4 | 58.0 | 60.1 | 58.2 | 57.9 | 56.2 | 58.8 | 55.0 |
| LFR (%) | 43.8 | 40.4 | 33.2 | 43.9 | 40.4 | 39.5 | 42.0 | 43.6 | 42.0 | 39.9 | 41.8 | 42.1 | 43.8 | 41.2 | 45.0 |
| MFR (%) | 20.9 | 17.2 | 22.0 | 22.0 | 21.0 | 18.6 | 16.9 | 18.1 | 19.9 | 16.9 | 19.3 | 20.5 | 17.3 | 16.9 | 16.3 |
| HFR (%) | 35.3 | 42.4 | 44.8 | 34.1 | 38.6 | 41.9 | 41.1 | 38.3 | 38.1 | 43.2 | 38.9 | 37.4 | 38.9 | 41.9 | 38.7 |
| RET-He (pg) | 18.7 | 18.7 | 18.7 | 17.9 | 18.2 | 18.5 | 18.6 | 18.4 | 18.1 | 18.8 | 18.7 | 18.2 | 18.6 | 19.1 | 18.2 |
| PLT (K/uL) | 844 | 1016 | 1125 | 946 | 1013 | 1248 | 1185 | 1057 | 1046 | 1068 | 1241 | 1031 | 1116 | 1106 | 1060 |
| PDW (fL) | 7.1 | 6.9 | 7.0 | 7.2 | 7.2 | 6.7 | 6.8 | 6.7 | 6.9 | 6.7 | 6.8 | 6.7 | 6.8 | 6.6 | 6.8 |
| MPV (fL) | 8.3 | 8.5 | 8.3 | 8.3 | 8.3 | 8.2 | 8.3 | 8.2 | 8.1 | 8.2 | 8.3 | 8.3 | 8.5 | 8.4 | 8.2 |
| P-LCR (%) | 4.1 | 3.4 | 2.1 | 3.1 | 3.1 | 3.2 | 2.5 | 3.0 | 3.3 | 1.9 | 4.2 | 3.4 | 3.2 | 2.8 | 3.0 |
| PCT (%) | 0.70 | 0.86 | 0.93 | 0.79 | 0.84 | 1.02 | 0.98 | 0.87 | 0.85 | 0.88 | 1.03 | 0.86 | 0.95 | 0.93 | 0.87 |
| WBC (K/uL) | 8.60 | 7.32 | 6.55 | 7.26 | 8.43 | 6.93 | 7.48 | 5.02 | 7.23 | 13.52 | 9.23 | 7.88 | 8.33 | 7.05 | 9.17 |
| NEUT (K/uL) | 0.98 | 0.71 | 0.81 | 4.17 | 0.75 | 1.20 | 0.67 | 0.44 | 0.66 | 1.05 | 0.82 | 0.87 | 0.82 | 0.83 | 0.79 |
| NEUT (%) | 11.4 | 9.8 | 12.3 | 57.4 | 9.0 | 17.4 | 9.0 | 8.8 | 9.1 | 7.7 | 8.8 | 11.0 | 9.9 | 11.9 | 8.6 |
| LYMPH (K/uL) | 7.41 | 6.42 | 5.61 | 2.95 | 7.54 | 5.55 | 6.66 | 4.49 | 6.43 | 12.23 | 8.22 | 6.89 | 7.30 | 6.10 | 8.17 |
| LYMPH (%) | 86.2 | 87.7 | 85.6 | 40.6 | 89.4 | 80.1 | 89.0 | 89.4 | 88.9 | 90.5 | 89.1 | 87.4 | 87.6 | 86.5 | 89.1 |
| MONO (K/uL) | 0.01 | 0.04 | 0.01 | 0.02 | 0.02 | 0.01 | 0.01 | 0.01 | 0.02 | 0.02 | 0.01 | 0.02 | 0.01 | 0.01 | 0.02 |
| MONO (%) | 0.1 | 0.5 | 0.2 | 0.3 | 0.2 | 0.1 | 0.1 | 0.2 | 0.3 | 0.1 | 0.1 | 0.3 | 0.1 | 0.1 | 0.2 |
| EO (K/uL) | 0.19 | 0.15 | 0.11 | 0.12 | 0.11 | 0.16 | 0.14 | 0.08 | 0.12 | 0.21 | 0.16 | 0.10 | 0.20 | 0.10 | 0.18 |
| EO (%) | 2.2 | 2.0 | 1.7 | 1.7 | 1.3 | 2.3 | 1.9 | 1.6 | 1.7 | 1.6 | 2.0 | 1.3 | 2.4 | 1.4 | 2.0 |
| BASO (K/uL) | 0.01 | 0.00 | 0.01 | 0.00 | 0.01 | 0.01 | 0.00 | 0.00 | 0.00 | 0.01 | 0.00 | 0.00 | 0.00 | 0.01 | 0.01 |
| BASO (%) | 0.1 | 0.0 | 0.2 | 0.0 | 0.1 | 0.1 | 0.0 | 0.0 | 0.0 | 0.1 | 0.0 | 0.0 | 0.0 | 0.1 | 0.1 |

Summary of complete blood count studies in vaccinated mice using serum one week after final vaccination

Abbreviations: RBC = Red Blood Cell Count; HGB = Hemoglobin value; HCT = Hematocrit value; MCV = Mean Corpuscular Volume; MHC = Mean Corpuscular Hemoglobin; MCHC = Mean Corpuscular Hemoglobin Concentration; RDW-SD = Red Cell Distribution Width Standard Deviation; RDW-CV = Red Cell Distribution Width Coefficient of Variation; RET = Reticulocytes; IRF =Immature Reticulocyte Fraction; LFR = Low Fluorescence Ratio; MFR = Medium Fluorescence Ratio; HFR = High Fluorescence Ratio; RET-He = Retic Hemoglobin; PLT = Platelet count; PDW = Platelet Distribution Width; MPV = Mean Platelet Volume ; P-LCR = Platelet Large Cell Ratio; PCT = Plateletcrit value; WBC = White Blood Cell Count; NEUT = Neutrophil percent; LYMPH = Lymphocyte percent; MONO = Monocyte; EO = Eosinophil; BASO = Basophil
