# Supplementary material for: Development of DNA Vaccine Targeting E6 and E7 Proteins of Human Papillomavirus 16 (HPV16) and HPV18 for Immunotherapy in Combination with Recombinant Vaccinia Boost and PD-1 Antibody
Source: mBio. 2021 Jan 19;12(1):e03224-20. doi: 10.1128/mBio.03224-20 (PMC7845631; doi:10.1128/mBio.03224-20)
Supplement: TABLE S5 [file mBio.03224-20-st005.docx]

**Table S5**: Summary of the biochemistry study in vaccinated mice

| Mouse Number | 1 | 2 | 3 | 4 | 5 | 6 | 7 | 8 | 9 | 10 | 11 | 12 | 13 | 14 | 15 | |
| --- | --- | --- | --- | --- | --- | --- | --- | --- | --- | --- | --- | --- | --- | --- | --- | --- |
| Test Day | 10/20/2020 | 10/20/2020 | 10/20/2020 | 10/20/2020 | 10/20/2020 | 10/20/2020 | 10/20/2020 | 10/20/2020 | 10/20/2020 | 10/20/2020 | 10/20/2020 | 10/20/2020 | 10/20/2020 | 10/20/2020 | 10/20/2020 | |
| Species | Mouse | Mouse | Mouse | Mouse | Mouse | Mouse | Mouse | Mouse | Mouse | Mouse | Mouse | Mouse | Mouse | Mouse | Mouse | |
| Strain | C57BL/6 | C57BL/6 | C57BL/6 | C57BL/6 | C57BL/6 | C57BL/6 | C57BL/6 | C57BL/6 | C57BL/6 | C57BL/6 | C57BL/6 | C57BL/6 | C57BL/6 | C57BL/6 | C57BL/6 | |
| Age (week) | 20 | 20 | 20 | 20 | 20 | 20 | 20 | 20 | 20 | 20 | 20 | 20 | 20 | 20 | 20 |  |
| Sex | Female | Female | Female | Female | Female | Female | Female | Female | Female | Female | Female | Female | Female | Female | Female | |
| Vaccination Group Number | DDV-1 | DDV-2 | DDV-3 | DDV-4 | DDV-5 | DDD-1 | DDD-2 | DDD-3 | DDD-4 | DDD-5 | PBS-1 | PBS-2 | PBS-3 | PBS-4 | PBS-5 | |
| ALB (g/dL) | - | - | - | - | - | - | - | - | - | 2.2 | 2.1 | 2.1 | 2.1 | 2.3 | - | |
| ALB/GLOB ratio | - | - | - | - | - | - | - | - | - | 0.71 | 0.64 | 0.64 | 0.60 | 0.77 | - | |
| ALP (U/L) | - | 155 | 155 | 155 | 185 | 142 | 155 | 146 | - | 137 | 115 | 125 | 141 | 152 | 143 | |
| ALT (U/L) | 29 | 33 | 34 | 35 | 37 | 26 | 38 | 31 | - | 33 | 33 | 27 | 30 | 29 | 31 | |
| AMY (U/L) | - | 1488 | 1578 | 1616 | 1667 | 1585 | - | 1688 | - | 1621 | 1478 | 1711 | 1491 | 1607 | 1444 | |
| AST (U/L) | 60 | 59 | 58 | 64 | 69 | 49 | - | 58 | - | 53 | 57 | 50 | 50 | 53 | 64 | |
| BUN (mg/dL) | 19 | 21 | 25 | 24 | 29 | - | - | 23 | - | 26 | 21 | 17 | 22 | 22 | - | |
| BUN/CREA ratio | 95.00 | 70.00 | 62.50 | 120.00 | 72.50 | - | - | 76.67 | - | 86.67 | 70.00 | 85.00 | 73.33 | 220.00 | - | |
| CA (mg/dL) | 9.4 | 10.1 | 10.1 | 12.4 | 10.6 | - | - | - | - | 10.0 | 9.8 | 9.7 | 10.1 | 10.2 | - | |
| CA (ALB) (mg/dL) | - | - | - | - | - | - | - | - | - | 11.3 | 11.2 | 11.1 | 11.5 | 11.4 | - | |
| CA (TP) (mg/dL) | - | - | - | - | - | - | - | - | - | 11.2 | 10.9 | 10.8 | 11.2 | 11.4 | - | |
| CA/PHOS ratio | - | 1.9 | 2.4 | 2.5 | 2.2 | - | - | - | - | 2.2 | 2.2 | 1.9 | 2.3 | 1.9 | - | |
| CHOL (mg/dL) | - | - | - | - | - | - | - | - | - | 61 | 62 | 63 | 62 | 60 | - | |
| CK (U/L) | - | 81 | 167 | 140 | 73 | 60 |  | 127 | - | 133 | 56 | 133 | 102 | 134 | - | |
| CL (mmol/L) | - | 111 | 111 | 112 | 110 | - | - | - | - | 114 | 107 | 102 | 107 | 111 | - | |
| CREA (mg/dL) | 0.2 | 0.3 | 0.4 | 0.2 | 0.4 | 0.2 |  | 0.3 | - | 0.3 | 0.3 | 0.2 | 0.3 | 0.1 | 0.2 | |
| GGT (U/L) | - | <0 | - | - | - | <1 | <1 | <0 | - | <0 | <0 | <0 | <0 | <0 | <0 | |
| GLOBULIN (g/dL) | - | - | - | - | - | - | - | - | - | 3.1 | 3.3 | 3.3 | 3.5 | 3.0 | - | |
| GLUC (mg/dL) | 178 | 185 | 249 | 216 | 227 | 158 | - | 181 | - | 175 | 194 | 184 | 167 | 167 | - | |
| K (mmol/L) | - | 7.4 | 7.5 | 8.3 | 7.5 | - | - | 7.4 | - | 7.0 | 7.6 | 7.3 | 6.8 | 7.7 | - | |
| LDH (U/L) | - | 652 | 577 | 678 | 586 | 542 | - | 680 | - | 543 | 767 | 571 | 530 | 677 | - | |
| MG (mg/dL) | - | 3.1 | 3.0 | 3.1 | 3.5 | - | - | - | - | 3.2 | 3.1 | 3.1 | 3.2 | 3.2 | - | |
| NA (mmol/L) | - | 144 | 151 | 147 | 174 | - | - | 144 | - | 152 | 141 | 141 | 139 | 145 | - | |
| NA/K ratio | - | 19.5 | 20.1 | 17.7 | 23.3 | - | - | 19.5 | - | 21.7 | - | 19.3 | 20.4 | 18.8 | - | |
| osmality (mosm/k) | - | 305.8 | 324.8 | 314.6 | 371.0 | - | - | 306.3 | - | 323.0 | - | 298.3 | 295.1 | 307.1 | - | |
| PHOS (mg/dL) | - | 5.3 | 4.2 | 5.0 | 4.8 | 5.8 | - | 4.9 | - | 4.5 | 4.5 | 5.1 | 4.3 | 5.3 | 5.1 | |
| TBIL (mg/dL) | 0.3 | 0.2 | 0.3 | 0.3 | 0.4 | 0.3 | - | 0.3 | - | 0.3 | 0.3 | 0.3 | 0.3 | 0.3 | - | |
| TP (g/dL) | - | - | - | - | - | 5.6 | - | 5.6 | - | 5.3 | 5.4 | 5.4 | 5.6 | 5.3 | 5.8 | |
| TRIG (mg/dL) | - | - | - | - | - | 36 | 46 | 35 | - | 41 | 76 | 52 | 43 | 67 | - | |
| UA (mg/dL) | - | - | 4.0 | 4.4 | 4.3 | 4.1 | - | 4.8 | - | 4.2 | 2.1 | 2.3 | 2.4 | 2.9 | - | |

Summary of biochemistry study in vaccinated mice using serum one week after final vaccination

Abbreviations: ALB = Albumin; ALB/GLOB = Albumin to Globulin; ALP = Alkaline phosphatase; ALT = Alanine Aminotransferase; AMY = Amylase; AST = Aspartate Aminotransferase; BUN = Blood Urea Nitrogen; BUN/CREA = Blood Urea Nitrogen to Creatinine; CA = Calcium; CA (ALB) = Calcium corrected for Albumin; CA (TP) = Calcium corrected for Total Protein; PHOS- Phosphate; CHOL = Cholesterol; CK = Creatine Kinase; CL = Chloride; CREA = Creatinine; GGT = Gamma- Glutamyl Transferase; GLUC = Glucose; K = Potassium; LDH = Lactic Acid Dehydrogenase; MG = Magnesium; NA = Sodium; TBIL = Total Bilrubin; TP = Total Protein; TRIG = Triglyceride; UA = Uric Acid

“ – “Represents data not available due to machine reading error and limited serum
