# Supplementary material for: Development of DNA Vaccine Targeting E6 and E7 Proteins of Human Papillomavirus 16 (HPV16) and HPV18 for Immunotherapy in Combination with Recombinant Vaccinia Boost and PD-1 Antibody
Source: mBio. 2021 Jan 19;12(1):e03224-20. doi: 10.1128/mBio.03224-20 (PMC7845631; doi:10.1128/mBio.03224-20)
Supplement: TABLE S6 [file mBio.03224-20-st006.docx]

**Table S6** Histological examination of key organs in mice receiving vaccination. (Mice were sacrificed on 10/20/2020).

| Vaccination Group | PBS | | | | |
| --- | --- | --- | --- | --- | --- |
| Mouse Vaccination Number: | PBS-1 | PBS-2 | PBS-3 | PBS-4 | PBS-5 |
| Date | 10/20/2020 | 10/20/2020 | 10/20/2020 | 10/20/2020 | 10/20/2020 |
| Brain | WNL/NSF | WNL/NSF | WNL/NSF | WNL/NSF | WNL/NSF |
| Thyroid | NT | NT | WNL/NSF | WNL/NSF | NT |
| Lung & Trachea | WNL/NSF | WNL/NSF | WNL/NSF | WNL/NSF | WNL/NSF |
| Trachea | WNL/NSF | WNL/NSF | WNL/NSF | WNL/NSF | WNL/NSF |
| Heart | WNL/NSF | WNL/NSF | WNL/NSF | WNL/NSF | WNL/NSF |
| Stomach | WNL/NSF | WNL/NSF | WNL/NSF | WNL/NSF | WNL/NSF |
| Small intestine | WNL/NSF | WNL/NSF | WNL/NSF | WNL/NSF | WNL/NSF |
| Large intestine | WNL/NSF | WNL/NSF | WNL/NSF | WNL/NSF | WNL/NSF |
| Pancreas | WNL/NSF | WNL/NSF | NT | WNL/NSF | WNL/NSF |
| Liver | WNL/NSF | WNL/NSF | WNL/NSF | WNL/NSF | WNL/NSF |
| Right kidney | WNL/NSF | WNL/NSF | WNL/NSF | WNL/NSF | WNL/NSF |
| Left kidney | WNL/NSF | WNL/NSF | WNL/NSF | WNL/NSF | WNL/NSF |
| Ovaries | WNL/NSF | WNL/NSF | WNL/NSF | WNL/NSF | WNL/NSF |
| Fallopian Tubes | NT | WNL/NSF | WNL/NSF | WNL/NSF | WNL/NSF |
| Uterus | WNL/NSF | WNL/NSF | WNL/NSF | WNL/NSF | WNL/NSF |
| Adrenal glands | WNL/NSF | WNL/NSF | WNL/NSF | WNL/NSF | WNL/NSF |
| Spleen | WNL/NSF | WNL/NSF | WNL/NSF | WNL/NSF | WNL/NSF |

WNL/NSF = Within normal limits / No significant findings (unremarkable)

NT = No Tissue

| Vaccination Group | DDD | | | | |
| --- | --- | --- | --- | --- | --- |
| Mouse Vaccination Number: | DDD-1 | DDD-2 | DDD- 3 | DDD- 4 | DDD- 5 |
| Date | 10/20/2020 | 10/20/2020 | 10/20/2020 | 10/20/2020 | 10/20/2020 |
| Brain | WNL/NSF | WNL/NSF | WNL/NSF | WNL/NSF | WNL/NSF |
| Thyroid | NT | WNL/NSF | NT | NT | WNL/NSF |
| Lung & Trachea | WNL/NSF | WNL/NSF | WNL/NSF | WNL/NSF | WNL/NSF |
| Trachea | WNL/NSF | WNL/NSF | NT | WNL/NSF | WNL/NSF |
| Heart | WNL/NSF | WNL/NSF | WNL/NSF | WNL/NSF | WNL/NSF |
| Stomach | WNL/NSF | WNL/NSF | WNL/NSF | WNL/NSF | WNL/NSF |
| Small intestine | WNL/NSF | WNL/NSF | WNL/NSF | WNL/NSF | WNL/NSF |
| Large intestine | WNL/NSF | WNL/NSF | WNL/NSF | WNL/NSF | WNL/NSF |
| Pancreas | WNL/NSF | WNL/NSF | WNL/NSF | WNL/NSF | WNL/NSF |
| Liver | WNL/NSF | WNL/NSF | WNL/NSF | WNL/NSF | WNL/NSF |
| Right kidney | WNL/NSF | WNL/NSF | WNL/NSF | WNL/NSF | WNL/NSF |
| Left kidney | WNL/NSF | WNL/NSF | WNL/NSF | WNL/NSF | WNL/NSF |
| Ovaries | WNL/NSF | WNL/NSF | WNL/NSF | WNL/NSF | WNL/NSF |
| Fallopian Tubes | NT | WNL/NSF | WNL/NSF | WNL/NSF | WNL/NSF |
| Uterus | WNL/NSF | WNL/NSF | WNL/NSF | WNL/NSF | WNL/NSF |
| Adrenal glands | WNL/NSF | WNL/NSF | WNL/NSF | WNL/NSF | WNL/NSF |
| Spleen | WNL/NSF | WNL/NSF | WNL/NSF | WNL/NSF | WNL/NSF |

WNL/NSF = Within normal limits / No significant findings (unremarkable)

NT = No Tissue

| Vaccination Group | DDV | | | | |
| --- | --- | --- | --- | --- | --- |
| Mouse Vaccination Number: | DDV-1 | DDV- 2 | DDV- 3 | DDV-4 | DDV-5 |
| Date | 10/20/2020 | 10/20/2020 | 10/20/2020 | 10/20/2020 | 10/20/2020 |
| Brain | WNL/NSF | WNL/NSF | WNL/NSF | WNL/NSF | WNL/NSF |
| Thyroid | WNL/NSF | WNL/NSF | NT | NT | WNL/NSF |
| Lung & Trachea | WNL/NSF | WNL/NSF | WNL/NSF | WNL/NSF | WNL/NSF |
| Trachea | WNL/NSF | WNL/NSF | WNL/NSF | WNL/NSF | WNL/NSF |
| Heart | WNL/NSF | WNL/NSF | WNL/NSF | WNL/NSF | WNL/NSF |
| Stomach | WNL/NSF | WNL/NSF | WNL/NSF | WNL/NSF | WNL/NSF |
| Small intestine | WNL/NSF | WNL/NSF | WNL/NSF | WNL/NSF | WNL/NSF |
| Large intestine | WNL/NSF | WNL/NSF | WNL/NSF | WNL/NSF | WNL/NSF |
| Pancreas | WNL/NSF | WNL/NSF | NT | WNL/NSF | WNL/NSF |
| Liver | WNL/NSF | WNL/NSF | WNL/NSF | WNL/NSF | WNL/NSF |
| Right kidney | WNL/NSF | WNL/NSF | WNL/NSF | WNL/NSF | WNL/NSF |
| Left kidney | WNL/NSF | WNL/NSF | WNL/NSF | WNL/NSF | WNL/NSF |
| Ovaries | WNL/NSF | WNL/NSF | WNL/NSF | WNL/NSF | WNL/NSF |
| Fallopian Tubes | WNL/NSF | WNL/NSF | WNL/NSF | WNL/NSF | WNL/NSF |
| Uterus | WNL/NSF | WNL/NSF | WNL/NSF | WNL/NSF | WNL/NSF |
| Adrenal glands | WNL/NSF | WNL/NSF | WNL/NSF | WNL/NSF | WNL/NSF |
| Spleen | WNL/NSF | WNL/NSF | WNL/NSF | WNL/NSF | WNL/NSF |

WNL/NSF = Within normal limits / No significant findings (unremarkable)

NT = No Tissue
